# Mobilizing community health assets through intersectoral collaboration for social connection: Associations with social support and well-being in a nationwide population-based study in Catalonia

**DOI:** 10.1371/journal.pone.0320317

**Published:** 2025-03-26

**Authors:** Pablo Galvez-Hernandez, Ketan Shankardass, Martine Puts, Ann Tourangeau, Luis Gonzalez-de Paz, Angelina Gonzalez-Viana, Carles Muntaner

**Affiliations:** 1 Lawrence Bloomberg Faculty of Nursing, University of Toronto, Toronto, Ontario, Canada; 2 Institute of Health Policy, Management and Evaluation, Dalla Lana School of Public Health, University of Toronto, Toronto, Ontario, Canada; 3 ICES Post-doctoral Trainee, Primary Care & Health Systems Research Program, University of Toronto, Toronto, Ontario, Canada; 4 Department of Health Sciences, Wilfrid Laurier University, Waterloo, Ontario, Canada; 5 MAP Centre for Urban Health Solutions, Li Ka Shing Knowledge Institute, St Michael’s Hospital, Toronto, Ontario, Canada; 6 Dalla Lana School of Public Health, University of Toronto, Toronto, Ontario, Canada; 7 Primary Healthcare Transversal Research Group, Institut d’Investigacions Biomèdiques August Pi i Sunyer (IDIBAPS), Barcelona, Spain; 8 Consorci d’Atenció Primària de Salut Barcelona Esquerra (CAPSBE), Barcelona, Spain; 9 Department of Public Health, Mental Health and Mother and Child Health, University of Barcelona, Barcelona, Spain; 10 Public Health Agency of Catalonia, Health Department, Barcelona, Spain; 11 Community-Oriented Primary Care Group, Catalan Society of Family and Community Medicine. Barcelona, Spain; 12 GREDS, and Johns Hopkins-UPF Public Policy Center, Department Ciencies Politiques & BSM-Barcelona School of Management, Universitat Pompeu Fabra Barcelona, Spain; University of Coimbra: Universidade de Coimbra, PORTUGAL

## Abstract

**Background:**

Limited social connection among older adults poses a global public health challenge, reducing sources of support and affecting health and well-being. National public health strategies that leverage local intersectoral collaboration between key sectors such as primary and social care, community organizations, and society, have been advocated, yet their impact remains underexplored.

**Objective:**

This study examines the regional variability in the uptake of a public health strategy in Catalonia that mobilizes community health assets, such as social clubs and leisure activities, through intersectoral collaboration and its associations with social support and mental well-being in older adults.

**Methods:**

We conducted a population-based cross-sectional study using the Catalan Health Survey (2017-2021) with 6011 adults aged ≥ 60 years across 31 Health Sectors. Survey data were linked with area-level uptake metrics, generated using data analytic techniques. Individuals were categorized into three uptake groups based on the number and territorial distribution of asset-based initiatives within their area of residence. Multilevel regressions tested associations with social support (OSSS-3) and mental well-being (SWEMWBS), controlling for individual, contextual, and temporal factors.

**Results:**

Participants’ average age was 74.1 years ±  10.0 with 53.3% women. From 2017 to 2021, 2312 asset-based initiatives were registered across Health Sectors, ranging from 0 to 342 per sector. Residing in sectors with the highest uptake of initiatives (>15 initiatives per 10,000 population) was associated with higher social support (β = .34, p < .01) and mental well-being scores (β =  1.11, p < .01).

**Conclusion:**

Residing in areas with greater health assets mobilized through intersectoral collaboration was associated with higher social support and well-being among older adults. This study represents one of the first national evaluations of an intersectoral strategy aimed at mitigating the mental health impacts of limited social networks. Future public health strategies should prioritize equitable access for inclusive benefits.

## Introduction

### Background

Older adults are susceptible to losing social ties due to multiple factors, including life events such as the loss of a spouse or friends, health and cognitive decline, ageism, and socioeconomic factors such as low income, lack of transportation, and limited social activities in the community [1–3]. The WHO Commission on Social Connection, established in 2024, revealed that 1 in 4 older adults experience social isolation globally [4]. Multiple constructs related to the lack of structural, functional, and quality dimensions of social relationships, are associated with an increased likelihood of morbidity and mortality [5]. For example, adults experiencing loneliness and social isolation have an increased likelihood of all-cause mortality of 26% to 32%, respectively [6], as well as a higher risk of cardiovascular and mental diseases and lower well-being in older adults [7–9]. Maintaining strong social ties with friends and family has long been understood to play a vital role in promoting good health, life satisfaction, and overall well-being as people grow older [10, 11].

Social support is a crucial mechanism derived from one’s social networks with a positive effect on health [12–14]. Through engaging in social relationships within these networks, individuals can obtain emotional, instrumental, and financial support [15]. Acting as a stress buffer, social support can mitigate the impact of stressful life events on health, either via supportive behaviors from others (received support) or the reassurance that help is accessible (perceived support) [16]. Thus, social support from meaningful connections alleviates loneliness and social isolation and is associated with better physical health in older adults [17,18]. High social support can reduce all-cause mortality by 45% and cardio-cerebrovascular mortality by 60% [19].

The theoretical and empirical relation between social support and mental well-being has motivated these constructs to be studied together [20–22]. Mental well-being is a multifaceted concept deeply intertwined with social interactions and can be defined as a state of positive mental health that encompasses life satisfaction and effective psychological functioning, including optimism, feeling useful and relaxed, clear thinking, and autonomy in decision-making [23]. Mental well-being is a strong predictor of older adult mortality [24] and is positively affected by participation in social activities [25,26]. Engaging in social activities can provide a sense of belonging, purpose, and mutual support, which are essential for maintaining mental well-being and, by extension, overall longevity and quality of life [25,26]. However, not all social ties within an individual’s social network contribute to social support and mental well-being, as this depends on the characteristics of the relationships, such as attachment, intimacy, and reciprocity, as well as the characteristics of the social network (e.g., size, density) [13,27]. Indeed, increasing opportunities for social contact with others is described as an effective strategy for promoting social networks in older adults, leading to benefits from associated positive mechanisms such as social support [28].

Strategies to promote social connection and support among older adults vary widely. Common approaches include individual-level interventions (e.g., one-to-one befriending [29]), group-based initiatives like group therapy [30] and peer support groups [31], and social activities to cultivate interpersonal interactions [32]. A recent review of initiatives to promote social connection through primary care, including social prescribing (i.e., healthcare professionals formally referring patients to non-clinical services), found that most interventions were limited to single settings [33]. In contrast, societal-level responses such as national public health strategies remain underexplored in the literature [34], despite the need for approaches that strengthen community infrastructure and address the complex factors underlying low social support [35].

A 2020 consensus report by the National Academies of Sciences, Engineering, and Medicine recommended strengthening ties between the healthcare system and community-based networks and resources for public health responses to tackle lack of social connection among older adults [36]. To implement this recommendation, sustained collaboration through partnerships across organizations from various health and non-health sectors is needed, a strategy known as intersectoral collaboration [37]. Such collaborative efforts are crucial for pooling resources (e.g., financial support, professional expertise) to address complex health and social issues that cannot be resolved independently by one sector alone [5,38]. For example, intersectoral collaboration can enhance the identification of isolated individuals through organizations from different sectors (e.g., primary care, social services, neighborhood associations), connect older adults to resources for social activity, provide follow-up support, and address social determinants such as inadequate housing or poverty, all tailored to the local context [36]. While intersectoral partnerships exist (e.g., the Los Angeles Social Isolation and Loneliness Impact Coalition connects media companies and non-profit retiree organizations, and Social Prescribing Programs link third-sector, community organizations and primary care settings in Canada, the UK, and Catalonia [39–41]), few studies assessed these systematic collaborations with healthcare systems across provinces or nations through intersectoral public health strategies, leaving an evidence gap [36].

Healthcare professionals, particularly in primary care, can be pivotal stakeholders in intersectoral collaborations [36,42], as isolated older adults frequently continue engaging with their family physicians, nurses and social workers [43]. Additionally, incorporating existing community health assets (e.g., social clubs, volunteering, and charity organizations) into intersectoral initiatives can enhance their sustainability and alignment with community culture [36,44].

Population-level studies have demonstrated the positive impact of social engagement and community participation among older adults [44,45], yet there is limited evidence on how large-scale public health strategies, particularly those involving intersectoral collaboration, relate to social support and mental well-being outcomes. One reason for the limited evaluation of national public health strategies is the challenge of capturing their area-level implementation, particularly for topics not typically covered by public databases like the census [46]. Limited capacity and lack of prioritization for continuous monitoring contribute to this gap, yet data analytic techniques like text mining and spatial analysis of web-scraped data might offer practical solutions for generating timely, localized datasets, enabling more rapid evaluations of population outcomes [47].

### Research context

Over the past decade, Catalonia, an autonomous province of Spain with approximately 8 million inhabitants and over 400 primary care centers, has pioneered intersectoral collaboration strategies [48], providing a unique context to explore existing gaps in the literature. In 2015, the Catalan Government launched the Inter-ministerial Public Health Plan (PINSAP) [49], a comprehensive strategy led by the Catalan Public Health Agency to strengthen intersectoral collaboration in addressing complex public health challenges at the governmental level. One of its main goals was to prevent social isolation and exclusion among older adults.

Two major programs under PINSAP (Community and Health and Social Prescribing and Health) were implemented locally through the establishment of intersectoral committees overseeing community health processes. The Community and Health program aimed to reorient healthcare toward health promotion, community engagement, and health equity, fostering public health policies tailored to local needs and resources [49]. The Social Prescribing and Health Program provided training, guidelines and educational materials for primary care professionals to strengthen their capacity to connect individuals with community resources, enhance their well-being, and reduce the risk of social isolation [40].

Local intersectoral committees, comprising primary care providers, public health professionals, community organization representatives, civil society groups, and municipal government officials, operated within local health jurisdictions, typically at the neighborhood or municipal level, and were nested within broader Health Sectors (also referred to as “regions” in this study). Local stakeholders were also equipped with the Finder of Assets and Health tool [50], a centralized open platform designed to map community health assets. This tool also facilitated the mobilization of these assets through intersectoral collaboration, enabling locally implemented referral and follow-up pathways and informal recommendations of assets that supported the two operational programs under the PINSAP umbrella.

The resulting asset-based initiatives include interventions, activities, and programs leveraging community assets (e.g., group leisure activities, adapted outdoor exercise, social retiree programs) to promote social interaction and well-being among older adults. These initiatives integrate linking pathways, which can be utilized by healthcare professionals, community organizations, municipal civil servants, and social services through social prescribing or other informal intersectoral community-based pathways. While Community and Health and Social Prescribing and Health outlined a framework for action, the uptake of these initiatives could vary significantly across regions, influenced by stakeholder engagement, local economic and political contexts, and existing collaborative networks [46].

### Objectives

This study aims to examine whether living in Health Sectors with greater uptake (defined by the number and territorial distribution of asset-based initiatives registered in the Assets and Health platform) is associated with higher levels of social support and better mental well-being. This study seeks to provide critical insights into the role of large-scale strategies that leverage community health assets through intersectoral collaboration to improve social engagement and mental well-being among older adults.

We hypothesize that: (1) older adults in regions with a higher number of intersectoral, asset-based initiatives will report greater social support and mental well-being compared to those in regions with fewer or no initiatives, and (2) those in regions with a broader territorial distribution of these initiatives will report higher social support and mental well-being compared to those in regions with less extensive distribution.

## Methods

### Study design, participants and data sources

We conducted a population-based cross-sectional study of five pooled waves (2017-2021) of the Catalan Health Survey (Enquesta de Salut de Catalunya [ESCA]), linked with data on the availability of asset-based initiatives to foster socialization among older adults across Health Sectors. Our study sample included a subset of 6011 respondents aged 60 and above from the five waves of the ESCA across all 31 Health Sectors in Catalonia.

Three data sources were used in this study. The ESCA is an annual, population-based household survey that assesses health status, behaviors, morbidity, quality of life, social determinants, health service utilization, and demographics in Catalonia [51]. Each year, a random representative sample of non-institutionalized individuals (living in their households), stratified by age, gender, and Health Sector, is drawn from the Catalan census [51]. The Health Department of Catalonia manages the survey in collaboration with the Statistical Institute of Catalonia, the University of Barcelona, and independent organizations for data validation. A Health Sector is considered a minimum sampling unit with an error of 5% and proportional weighting to ensure comparable indicators across waves. Each wave of the ESCA typically samples 4800 individuals. Data collection is conducted face-to-face by trained personnel throughout the year (January to December), avoiding repetition of the same census units to ensure internal variability and allowing the pooling of cross-sectional waves [52].

Datasets on regional health assets, mapped and integrated into intersectoral collaboration networks, were generated from the Assets and Health platform. Detailed descriptions of each community health asset, including title, description, target population, and location, are stored on independent dedicated websites linked to the platform [50]. We combined web scraping, text mining, and spatial overlay analysis to extract, classify, and geolocate web-based text data on health assets from 50,000 websites within the Finder of Assets and Health. Eligible health assets had to target older adults as the primary population and consist of either group activities (e.g., walking groups with primary care and social services referrals) or individual activities designed to enhance social connections (e.g., volunteer-based befriending involving social services and retiree associations). The process of health asset dataset creation is detailed in our previous publication [47].

The third data source was the Community Health Indicators, a public database from the Catalan Health System Observatory, containing 38 small-area demographic and socioeconomic indicators [53]. These indicators, available for 2016 and 2018, aggregate individual data from various public institutions at the area level, such as the census and primary healthcare services, to facilitate local health diagnostics for local community health processes.

### Variables and measures

#### Dependent variables.

Social support and mental well-being were extracted from the ESCA. Social support was measured using the 3-item Oslo Social Support Scale (OSSS-3) [54], which examines the number of close individuals the respondent can rely on, the perceived interest and concern from others, and the perceived availability of practical assistance from neighbours. This scale has moderate reliability (Cronbach’s alpha = .640) and has been applied in several large-scale population surveys across different settings, including the Eurobarometer and the European Health Survey [51]. The items have a Likert-scale format yielding a score of 3 to 14, with higher scores indicating higher perceived social support. Mental well-being was measured through the 7-item Warwick-Edinburgh Mental Well-being Scale (SWEMWBS) [55], which captures affective-emotional aspects of well-being, cognitive-evaluative dimensions, and psychological functioning, and has demonstrated reliable internal consistency (Cronbach’s alpha =  0.84) [55]. The score ranges from 7 to 35, with higher scores indicating better mental well-being [56].

#### Regional uptake.

Regional uptake was measured through two variables. First, “number of initiatives” quantified eligible asset-based initiatives (those targeting older adults, group-based, or individual initiatives such as befriending) registered by local stakeholders on the Assets and Health platform in each Health Sector annually from 2017 to 2021. This variable was categorized into three groups based on the cumulative number of initiatives per 10,000 people: no uptake (0 initiatives), below the median (1-15 initiatives), and above the median (>15 initiatives). Second, “territorial distribution” captured how widely initiatives were spread across smaller sub-areas within each Health Sector. We categorized Health Sectors into three groups based on the percentage of their sub-areas that had at least one initiative: no uptake, ≤ 50%, and > 50% of sub-areas.

#### Individual-level covariates.

Participants self-reported sociodemographic covariates extracted from the ESCA included: age (measured in years), sex (man, woman), education (categorized according to the International Standard Classification of Education into two levels: primary studies or less, secondary studies or higher), employment status (active, retired or unemployed, domestic work), economic strain as self-reported insufficient economic resources to make ends meet (no economic strain, economic strain), nationality (Spanish, non-Spanish), and household size (number of individuals living together). Health and functional status variables associated with lower social support and mental well-being were also included as covariates [57,58]: limitations for daily activities (no limitation, not severe limitation, severe limitation) and number of chronic conditions from a list of 30 conditions listed in the ESCA survey. A time variable spanning survey years from 2017 to 2021 was included in the analysis to control for temporal changes in social support and mental well-being.

#### Contextual-level covariates.

Variables extracted from the Community Health Indicators database included the Health Sectors of residence, Health Sector population, proportion of people over 75 years living alone in each Health Sector, and the socioeconomic index - a composite socioeconomic measure of unemployment, low educational level, immigration, and low income [59]. These variables accounted for the cultural, economic, and demographic heterogeneity across different Health Sectors that might be not explained by individual-level factors.

### Data analysis

Data integration involved the following steps: Data from the five ESCA waves (2017-2021) were pooled into a single dataset. We selected waves from 2017 onwards since uniform measurement scales for social support and mental well-being were introduced that year. Subsequently, we linked the dataset on regional uptake of initiatives with ESCA data, assigning each participant to uptake groups based on the number and territorial distribution of initiatives in their Health Sector of residence during the year they participated in the ESCA survey. Finally, contextual-level covariates were linked to each observation based on the Health Sector of residence using 2018 Community Health Indicators data. Participants in Health Sectors with no uptake served as the reference group. This approach aligns with methodologies used in other population-based survey evaluations [60].

Descriptive statistics were calculated for dependent variables and individual-level covariates. Bivariate analyses, Bonferroni-adjusted for multiple hypothesis testing, examined relationships between covariates and dependent variables. Parametric tests (Pearson’s correlation, t-test, ANOVA) were conducted and compared with non-parametric tests to ensure robustness in the presence of potential outliers.

To test our hypotheses, multilevel linear regression models with a random intercept were developed to account for the nested nature of participants within Health Sectors, controlling for covariates at both individual and contextual levels [61]. Social support and mental well-being were included as continuous dependent variables based on OSSS-3 and SWEMWBS scale scores. Initial models included dependent and regional uptake variables controlled by survey year, and Health Sectors (Model 1). Subsequent models added individual-level covariates (Model 2), and contextual covariates (Model 3). A parsimonious Model 4 was derived from Model 3 through a manual backward stepwise approach, optimizing for goodness of fit using the Akaike Information Criterion (AIC) and a p-value cutoff of 0.10 [62]. Four iterations of Models 1 to 4 were developed to independently test associations for each combination of dependent variable (social support, mental well-being) and regional uptake variable (number of initiatives, territorial distribution). All analyses were performed using RStudio (Version 2023.12.1) [63] with Maximum Likelihood estimation using the “lmer.test” library [64] at an α level of.05. Marginal and conditional R^2^ were computed to examine the variability in social support and mental well-being explained by fixed and random effects. Regression assumptions were checked through visualization and Variance Inflation Factors (VIF) for multicollinearity [65].

Out of 6011 observations, 1000 (16.6%) had missing data, mostly social support (n = 783) and mental well-being (n = 621). Missing values of social support and mental well-being were correlated with age (*r* = 0.29, *p* > 0.001) and higher chronic conditions (*r* = 0.14, *p* > 0.001), denoting Data Missing at Random (MAR). We estimated missing values using the “mice” package in R [66] using predictive mean matching for continuous variables and logistic regression for categorical variables [67].

Several sensitivity analyses were performed to test model assumptions. First, we repeated the analyses without imputing missing data and compared single-level linear models with Health Sector as a fixed effect to the original models. To assess linearity and normality assumptions, we repeated the analysis using robust multilevel models to adjust for potential outliers [68]. We also reanalyzed the data by categorizing social support and mental well-being based on scale cutoffs. For social support, we used Ordinal Logistic Mixed Models (CLMM) [69] to calculate odds ratios for poor (OSSS-3 scores 3-8), moderate (9-11), and strong (12-14) social support [52] by regional uptake. For mental well-being, we used Generalized Linear Mixed-Effects Models (GLMM) with dichotomous outcomes: low (SWEMWBS score < 26) and good (≥26) well-being [53]. Specificity analyses involved repeating the models with the number of chronic conditions as the dependent variable. To assess potential biases related to temporal and age-specific effects, we excluded data from the COVID-19 period (2020-2021) and reanalyzed a subset of participants aged > 65. Finally, a post hoc analysis examined the potential moderating effect of social support on mental well-being by including an interaction term between social support and the total number of initiatives [12].

### Ethics approval

This research was approved by the Health Sciences Research Ethics Board of the University of Toronto on November 11, 2022 (reference number #43639). The authors did not have access to any personal information that could identify individual participants. No ethical consent was required as the data were de-identified and anonymized. The use of the Catalan Health Survey (ESCA) is authorized under the current Statistical Plan of Catalonia, which guarantees the protection of personal data (Article 36 of Law 23/1998, of December 30, on Statistics of Catalonia).

## Results

### Descriptive analysis

Among the 6011 participants, the average age was 74.11 years (SD 10.01), and 53.3% were women. As shown in Table 1, the mean social support score (OSSS-3) was 10.93 (SD 1.86) out of 14, indicating an average moderate social support. The mean well-being (SWEMWBS) was 28.29 (SD 5.96) out of 35, corresponding to an average good mental well-being.

**Table 1:** Sociodemographic characteristics of adults aged > 60 years in Catalonia (N = 6011) from the Catalan Health Survey (2017-2021) and bivariate analysis with variables.

			OSSS-3^a^		SWEMWBS	
Variables	n	%	r/F/*t*^*b*^	p-value	r/F/*t*	p-value
**Individual-level covariates**						
Age in years, Mean (SD)	74.11	10.01	-0.02	0.113	-0.20	<0.001 ^*^
Number of household members, Mean (SD)	2.17	0.97	0.04	0.003	0.02	0.197
Number of chronic conditions, Mean (SD)	4.27	3.14	-0.08	<0.001 ^*^	-0.35	<0.001 ^*^
Sex			1.56	0.119	10.88	<0.001 ^*^
Male	2806	46.7				
Female	3205	53.3				
Limited in basic activities of daily living			26.37	<0.001 ^*^	597.2	<0.001 ^*^
Severe limitation	619	10.3				
Limited, not severe	1335	22.2				
Not limited	4057	67.5				
Education			-1.62	0.105	-15.66	<0.001 ^*^
Primary school or less	2610	43.4				
Secondary school or higher	3401	56.6				
Employment			3.22	<0.05	74.31	<0.001 ^*^
Active employment	711	11.8				
Retired or unemployed	4215	70.1				
Domestic work	1085	18.1				
Economic strain			-4.74	<0.001 ^*^	-12.73	<0.001 ^*^
No	5019	83.5				
Yes	992	16.5				
Nationality			4.98	<0.001 ^*^	-1.85	0.066
Spanish	5824	96.6				
Non-Spanish	187	3.11				
**Regional uptake**						
Number of initiatives per 10,000 population			6.11	<0.01	6.14	<0.01
No uptake	1221	20.3				
1-15	3558	59.2				
>15	1232	20.5				
Territorial distribution			4.77	0.092	.75	0.474
No uptake	1221	20.3				
≤50%	2935	48.8				
>50%	1855	30.9				
**Dependent variables**						
OSSS-3, Mean (SD)	10.93	1.86	–	–	0.31	<0.001 ^*^
SWEMWBS, Mean (SD)	28.29	5.96	0.31	<0.001 ^*^	–	–

^a^OSSS-3 =  3-item Oslo Social Support Scale; SWEMWBS =  Warwick-Edinburgh Mental Well-being Scale.

^b^r =  Pearson correlation coefficient; F =  One-way ANOVA F statistic; *t* = t-test statistic.

* Significant p- values at the Bonferroni-adjusted significance level of 0.002 (α=.05/23 tests).

For the period 2017-2021, 2312 eligible initiatives registered in the Assets and Health platform were identified, ranging from 0 to 342 cumulative initiatives across Health Sectors, with a standardized median of 15 initiatives per 10,000 population per Health Sector. Most initiatives registered by local stakeholders involved in intersectoral collaboration networks were focused on leisure or skill development activities (n = 987, 42.7%) and group physical activities (n = 674, 29.1%).. Examples included group handcrafts, theater, cooking, choir courses, walking groups, and outdoor gym activities, to which older adults could be linked through entities from primary care, social and community services, municipal government, and neighborhood organizations. Characteristics and definitions of the initiatives and their uptake across Health Sectors are provided in S1 File.

**Fig 1:**
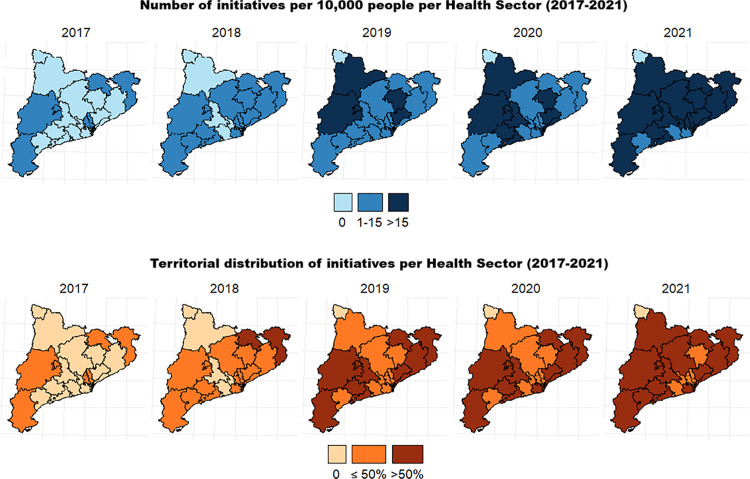
Regional uptake based on two variables: total number and territorial distribution of asset-based initiatives registered by stakeholders in intersectoral collaboration networks across Health Sectors (2017-2021).

About 23% of participants (n = 1221) lived in Health Sectors with no uptake at some point between 2017 and 2021, and 59.2% (n = 3558) lived in areas with 1-15 initiatives per 10,000 population. Additionally, 48.8% of older adults (n = 2935) lived in Health Sectors where initiatives were available in less than 50% of the sub-areas. Fig 1 illustrates regional uptake, with the Health Sector boundaries from the Catalan Government’s open-source database as the geographical layer, and a second layer representing the regional uptake of initiatives.

In bivariate analyses with Bonferroni adjustment (*p* < 0.002), variables denoting regional uptake of the public health strategy were not significantly associated with older adults’ social support or mental well-being scores. Significant negative associations were found for higher number of chronic conditions, experiencing severe limitations in daily activities, economic strain, non-Spanish status, and lower social support scores. Additionally, higher age, a greater number of chronic conditions, being female, having severe daily activity limitations, lower education levels, domestic work or unemployment, and economic strain were negatively associated with mental well-being (Table 1).

### Multilevel linear regression models

#### Social support.

All adjusted multilevel models (Models 1 to 4) controlled for demographic, socioeconomic, health and temporal covariates, showed statistically significant positive associations between residing in Health Sectors with higher uptake of initiatives and OSSS-3 social support scores. Older adults in Health Sectors with > 15 initiatives per 10,000 population had higher estimated average social support scores (β= 0.34, 95% CI =  0.07- 0.60, *p* = 0.013) than those in sectors without initiatives, with lower but positive coefficients for those living in areas with 1-15 activities (β=0.25, 95% CI =  0.05-0.44, *p* = 0.012), showing a stepwise pattern. Similar patterns were observed for older adults residing in Health Sectors with a greater territorial distribution of initiatives (Table 2).

**Table 2:** Multilevel linear regression models estimating the association between social support and regional uptake variables: number and territorial distribution of asset-based initiatives registered in Health Sectors. Coefficients represent the estimated average differences in OSSS-3 social support scores (N = 6011).

	Model 1	p-value	Model 2	p-value	Model 3	p-value	Model 4	p-value
	β Coefficient (95% CI)		β Coefficient (95% CI)		β Coefficient (95% CI)		β Coefficient (95% CI)	
**Intercept**	11.15 (10.99 – 11.30)	<0.001	11.38 (11.16 – 11.61)	<0.001	11.37 (11.14 – 11.59)	<0.001	11.30 (11.14 – 11.45)	<0.001
**Number of initiatives per 10,000 population**								
No uptake	ref		ref		ref		ref	
1-15 initiatives	0.25 (0.05 – 0.44)	0.013	0.25 (0.05 – 0.44)	0.013	0.26 (0.07 – 0.46)	0.009	0.25 (0.05 – 0.44)	0.012
> 15 initiatives	0.32 (0.06 – 0.59)	0.018	0.34 (0.07 – 0.60)	0.014	0.37 (0.10 – 0.64)	0.008	0.34 (0.07 – 0.60)	0.013
**Intercept**	11.15 (10.99 – 11.30)	<0.001	11.38 (11.15 – 11.60)	<0.001	11.36 (11.13 – 11.59)	<0.001	11.29 (11.14 – 11.45)	<0.001
**Territorial distribution**								
No uptake	ref		ref		ref		ref	
≤ 50%	0.25 (0.05 – 0.44)	0.015	0.25 (0.05 – 0.44)	0.013	0.26 (0.07 – 0.46)	0.009	0.25 (0.05 – 0.44)	0.012
> 50%	0.28 (0.02 – 0.54)	0.037	0.31 (0.05 – 0.57)	0.019	0.35 (0.08 – 0.62)	0.010	0.32 (0.06 – 0.58)	0.018

Model 1 =  main independent variable, survey year, and Health Sectors; Model 2 =  main independent variable, controlled for individual covariates, survey year, and Health Sectors; Model 3 =  same variables plus context-level covariates; Model 4 =  parsimonious model.

As shown in Table 3, individual-level covariates remained significant in the adjusted multilevel models, with negative associations similar to those in the bivariate analysis. Being non-national had the largest negative effect on social support (β =  -0.87, 95% CI =  -1.13 to -0.60; *p* < 0.001). Residing in households with more members became significantly positively associated with social support scores after adjusting for individual and contextual covariates (β =  0.10, 95% CI =  0.06 to 0.15; *p* < 0.001). Survey year coefficients showed a declining trend in older adults’ social support scores from 2018 through 2020, with a recovery noted in 2021. No significant associations were found for context-level covariates. Fixed effects accounted for 3.5% of the variability in social support, which increased to 6% after including random intercepts (Conditional R² =  0.060). The complete outputs for Models 1 to 4 on social support including random effects estimates are provided in S2 File.

**Table 3:** Parsimonious multilevel regression model for social support, including regional uptake variables, adjusted for individual and contextual covariates. Coefficients denote the estimated average differences in OSSS-3 social support scores (N = 6011).

	Model 4a	p-value	Model 4b	p-value
**Fixed effects**	β Coefficient (95% CI)		β Coefficient (95% CI)	
**Intercept**	11.30 (11.14 – 11.45)	<0.001	11.29 (11.14 – 11.45)	<0.001
**Regional uptake**				
Number of initiatives per 10,000 population				
No uptake	ref			
1-15 initiatives	0.25 (0.05 – 0.44)	0.012		
> 15 initiatives	0.34 (0.07 – 0.60)	0.013		
Territorial distribution				
No reach			ref	
≤ 50%			0.25 (0.05 – 0.44)	0.012
> 50%			0.32 (0.06 – 0.58)	0.018
**Individual-level covariates**				
Number of chronic conditions	-0.05 (-0.06 – -0.03)	<0.001	-0.05 (-0.06 – -0.03)	<0.001
Limitation in daily activities				
No limitation	ref		ref	
Limitation, not severe	-0.06 (-0.18 – 0.06)	0.358	-0.06 (-0.18 – 0.06)	0.369
Severe limitation	-0.36 (-0.53 – -0.19)	<0.001	-0.36 (-0.53 – -0.19)	<0.001
Number of household members	0.10 (0.06 – 0.15)	<0.001	0.10 (0.06 – 0.15)	<0.001
Economic strain				
No	ref		ref	
Yes	-0.18 (-0.31 – -0.05)	0.007	-0.18 (-0.31 – -0.05)	0.007
Nationality				
Spanish	ref		ref	
Non-Spanish	-0.87 (-1.13 – -0.60)	<0.001	-0.86 (-1.13 – -0.59)	<0.001
**Survey year**				
2017	ref		ref	
2018	-0.46 (-0.64 – -0.28)	<0.001	-0.46 (-0.64 – -0.28)	<0.001
2019	-0.77 (-0.98 – -0.57)	<0.001	-0.78 (-0.99 – -0.56)	<0.001
2020	-0.74 (-0.97 – -0.51)	<0.001	-0.74 (-0.98 – -0.50)	<0.001
2021	-0.47 (-0.70 – -0.25)	<0.001	-0.47 (-0.70 – -0.23)	<0.001

Model 4a = parsimonious model for social support and number of initiatives per 10,000 population in the Health Sector of residence. Model 4b = parsimonious model for social support and territorial distribution of initiatives.

### Mental well-being

Model 1 (controlled for the temporal variable survey year and Health Sector) showed no statistically significant associations between regional uptake variables and mental well-being scores. After controlling for socioeconomic and health-related individual covariates (Models 2 to 4), living in Health Sectors with 1 to 15 initiatives per 10,000 population became positively associated with mental well-being scores (β= 0.59, 95% CI =  0.04-1.14, *p* = 0.036), with even higher estimates for older adults living in sectors with > 15 initiatives per 10,000 population (β=1.11, 95% CI =  0.36-1.86, *p* = 0.004) (Table 4). Territorial distribution was not associated with mental well-being scores.

**Table 4:** Multilevel linear regression models estimating the association between mental well-being and regional uptake variables: number and territorial distribution of asset-based initiatives registered in Health Sectors. Coefficients represent the estimated average differences in SWEMWBS mental well-being scores (N = 6011).

	Model 1	p-value	Model 2	p-value	Model 3	p-value	Model 4	p-value
	Coefficient (95% CI)		Coefficient (95% CI)		Coefficient (95% CI)		Coefficient (95% CI)	
**Intercept**	28.34 (27.88 – 28.80)	<0.001	29.80 (29.17 – 30.43)	<0.001	29.79 (29.15 – 30.43)	<0.001	29.82 (29.34 – 30.30)	<0.001
**Number of initiatives per 10,000 population**								
No uptake	ref		ref		ref		ref	
1-15 initiatives	0.40 (-0.22 – 1.03)	0.208	0.58 (0.03 – 1.13)	0.040	0.59 (0.03 – 1.14)	0.040	0.59 (0.04 – 1.14)	0.036
> 15 initiatives	0.84 (-0.01 – 1.70)	0.054	1.10 (0.35 – 1.86)	0.004	1.11 (0.34 – 1.87)	0.005	1.11 (0.36 – 1.86)	0.004
**Intercept**	28.33 (27.85 – 28.80)	<0.001	29.78 (29.14 – 30.42)	<0.001	29.78 (29.13 – 30.43)	<0.001	29.81 (29.31 – 30.30)	<0.001
**Territorial distribution**								
No uptake	ref		ref		ref		ref	
≤ 50%	0.33 (-0.30 – 0.96)	0.302	0.53 (-0.03 – 1.08)	0.064	0.52 (-0.04 – 1.09)	0.070	0.54 (-0.02 – 1.10)	0.058
> 50%	-0.16 (-1.00 – 0.68)	0.717	0.33(-0.41 – 1.08)	0.381	0.32 (-0.44 – 1.08)	0.409	0.34 (-0.40 – 1.09)	0.367

Model 1 = main independent variable, survey year, and Health Sectors; Model 2 =  main independent variable, controlled for individual covariates, survey year, and Health Sectors; Model 3 =  same variables plus context-level covariates; Model 4 =  parsimonious model.

All negative associations between individual-level covariates and mental well-being identified in the bivariate analysis remained significant in the adjusted multilevel models (Table 5), with large negative coefficients observed for being female (β =  -0.90, 95% CI =  -1.17 to -0.64; *p* < 0.001) and experiencing economic strain (β =  -1.13, 95% CI =  -1.51 to -0.76; *p* < 0.001). The strongest negative associations were found between lower mental-well being scores and severe limitations in daily activities (β =  -4.91, 95% CI =  -5.41 to -4.42; *p* < 0.001). Older adults with secondary or higher education had higher estimated mental well-being scores compared to those with primary education or less (β =  0.75, 95% CI 0.46 to 1.05; *p* < 0.001). Survey year coefficients indicated a reduction in mental well-being from 2018 onwards (Table 5). The parsimonious multilevel model’s individual fixed effects accounted for 23% of the variation in mental well-being scores, with an additional 2% captured by adding Health Sectors as random intercepts (Conditional R² =  0.25). Full outputs of Models 1 to 4 for mental well-being with random effects estimates are available in S2 File.

**Table 5:** Parsimonious multilevel regression model for mental well-being, including regional uptake variables, adjusted for individual and contextual covariates. Coefficients denote the estimated average differences in SWEMWBS mental well-being scores (N = 6011).

	Model 4a	p-value	Model 4b	p-value
**Fixed effects**	Coefficient (95% CI)		Coefficient (95% CI)	
**Intercept**	29.82 (29.34 – 30.30)	<0.001	29.81 (29.31 – 30.30)	<0.001
**Regional uptake**				
Number of initiatives per 10,000 population				
No uptake	ref			
1-15 initiatives	0.59 (0.04 – 1.14)	0.036		
> 15 initiatives	1.11 (0.36 – 1.86)	0.004		
Territorial distribution				
No uptake			ref	
≤ 50%			0.54 (-0.02 – 1.10)	0.058
> 50%			0.34 (-0.40 – 1.09)	0.367
**Individual-level covariates**				
Age	-0.03(-0.05 – -0.02)	<0.001	-0.03 (-0.05 – -0.02)	<0.001
Sex				
Man	ref		ref	
Woman	-0.90 (-1.17 – -0.64)	<0.001	-0.90 (-1.17 – -0.64)	<0.001
Number of chronic conditions	-0.38 (-0.43 – -0.33)	<0.001	-0.38 (-0.43 – -0.33)	<0.001
Limitation in daily activities				
No limitation	ref		ref	
Limitation, not severe	-2.16 (-2.51 – -1.81)	<0.001	-2.14 (-2.49 – -1.79)	<0.001
Severe limitation	-4.92 (-5.42 – -4.43)	<0.001	-4.91 (-5.41 – -4.42)	<0.001
Education				
Primary or less	ref		ref	
Secondary or higher	0.77 (0.47 – 1.06)	<0.001	0.75 (0.46 – 1.05)	<0.001
Economic strain				
No	ref		ref	
Yes	-1.13 (-1.50 – -0.76)	<0.001	-1.13 (-1.51 – -0.76)	<0.001
**Survey year**				
2017	ref		ref	
2018	-1.07 (-1.58 – -0.55)	<0.001	-1.03 (-1.55 – -0.51)	<0.001
2019	-1.11 (-1.70 – -0.52)	<0.001	-0.88 (-1.49 – -0.27)	0.005
2020	-1.26 (-1.92 – -0.60)	<0.001	-0.96 (-1.65 – -0.28)	0.006
2021	-1.23 (-1.87 – -0.59)	<0.001	-0.76 (-1.43 – -0.10)	0.025

Model 4a = parsimonious model for mental well-being and number of initiatives per 10,000 population in the Health Sector of residence. Model 4b = parsimonious model for mental well-being and territorial distribution of initiatives.

### Sensitivity analyses

Sensitivity analyses yielded consistent regression coefficients for both dependent variables under various conditions: non-imputed missing data, inclusion of Health Sector as a fixed effect, handling of individual-level and random effects outliers, and reanalysis of the population aged over 65 years. No multicollinearity among predictors was detected. Analyses with social support and mental well-being as categorical variables showed consistent stepwise effects. Older adults in Health Sectors with highest uptake ( > 15 initiatives per 10,000 population) had 1.66 times the odds of reporting high social support (OR =  1.66, 95% CI =  1.25 to 2.19, *p* < 0.001) and 1.41 times the odds of good mental well-being (OR =  1.41, 95% CI =  0.99 to 1.99, *p* = 0.054), compared to those in regions with no uptake. Excluding data from 2020-2021 to assess the impact of COVID-19 increased the coefficients’ magnitude for both dependent variables. The post hoc analysis revealed no significant interaction effect between the number of initiatives per 10,000 population and social support on mental well-being. All sensitivity analyses are detailed in S3 File.

## Discussion

This study examined the association between regional uptake of a national public health strategy in Catalonia, aimed at fostering community health, intersectoral collaboration and the mobilization of existing community health assets to enhance socialization, and its impact on older adults’ social support and mental well-being. To our knowledge, this is the first study to combine web-scraped data, text mining, and spatial overlay analysis with individual-level survey data to measure associations between area-level uptake of a public health strategy and individual health and social outcomes using multilevel analysis. Measuring regional uptake by the number of initiatives registered per Health Sector in the Assets and Health platform proved to be an efficient method for capturing area-level variability, offering a practical approach for program evaluation in contexts with limited capacity for continuous active monitoring. Moreover, our study is one of the first to evaluate the national uptake of such strategies in a representative sample of older adults, contributing to the limited multi-site literature on social connection constructs, which have primarily demonstrated benefits in reducing loneliness [70,71]. For instance, the UK’s social prescribing model, led by the British Red Cross in 37 sites, showed significant pre-post reductions in loneliness among adults [70]. Similarly, the Urban Health Centres Europe (UHCE) project in five European cities demonstrated positive effects on loneliness, though the impact on mental well-being was less clear [71].

Our results suggest that residents in Health Sectors with greater uptake of the strategy - where local stakeholders mapped and registered a higher number of community health assets for social engagement within intersectoral collaboration networks - had significantly higher estimated social support and mental well-being compared to those in areas with no uptake. A broader reported distribution of these asset-based initiatives within Health Sectors was positively associated with social support, but not with mental well-being. These associations remained consistent after adjusting for individual and contextual-level covariates, demonstrating their robustness.

Notably, we identified over 2300 eligible asset-based initiatives registered by stakeholders from 2017 to 2021, reflecting a significant increase of uptake over time. Our previous analyses showed that after mid-2021, there was a marked rise in asset-based initiative registrations [47], potentially linked to the expanded implementation of operational programs such as the Social Prescribing and Health Program [40]. However, these initiatives were unevenly available across Catalonia. For instance, the Health Sector with the highest uptake experienced substantial growth over time, with initiatives increasing from 4 in 2017 to 342 (cumulative absolute count) by 2021, while other sectors did not register any initiatives or started doing so later in the study period. Similarly, territorial distribution was reached in all sub jurisdictions of some Health Sectors by 2019, while others exhibited more gradual increases. This variability highlights an unequal pace of implementation across regions, likely influenced by determinants such as local resources, sociodemographic characteristics, stakeholder involvement, contextual barriers, and organizational culture [47].

Older adults in Health Sectors without registered initiatives may still access community group activities, but these assets are likely not integrated into a network that facilitates utilization by health and non-health sector organizations. Integrating community assets into intersectoral collaboration schemes can enhance the dissemination of interventions by organizations within each sector (e.g., primary care, third sector, social services) to older adults who could benefit from them [36,72]. Additionally, leveraging existing health assets in older adults’ residential areas can align initiatives with the cultural norms of each context, facilitating new social networks and potentially increasing their social support [73.^74^]. Previous studies have shown that formal recommendations of community assets by primary care professionals (e.g., social prescribing), social workers, or municipal civil servants are seen as trustworthy, reducing perceived barriers to participation [33]. As Tierney and colleagues’ realist review suggests, when local stakeholders collaboratively map community health assets and are aware of available resources and population needs, they can select initiatives that effectively enhance social connection [75]. Policymakers and public health implementers should prioritize territorial equity in social engagement opportunities for older adults by adopting implementation approaches that promote a shared vision and common language of intersectoral collaboration and community-based strategies among local stakeholders [76].

A key finding of our study is that older adults in Health Sectors with the highest uptake (>15 initiatives per 10,000 population) had the highest estimated social support and mental well-being scores, even after adjusting for individual, contextual-level and temporal covariates. A greater number of widely available asset-based initiatives in their place of residence may enhance access and utilization. Beyond the benefits of intersectoral collaboration in promoting access to initiatives and participant follow-up, previous research on the relationship between geographical availability and access suggests that living near social community resources favors social participation among older adults [77]. This is especially true for older adults, who are more place-bound and spend more time in their neighborhood, remaining in the same residential environment longer than the working population [78].

For individuals accessing asset-based initiatives, characteristics such as their group-based structure, frequent recurrence, and focus on participants with similar profiles may foster psychosocial mechanisms linked to higher levels of perceived social support [36]. Expanding the social networks through social engagement of older adults creates opportunities for connections with others, serving as a source of perceived support that may extend beyond their active participation in the initiatives [79]. An increased social network might facilitate older adults developing a wider range of social roles beyond family and relatives, which has been linked to higher scores of perceived social support [80].

The observed association between higher estimated mental well-being scores in older adults and residing in Health Sectors with a high uptake may have distinct explanations. Previous literature showed that engaging in social activities with other participants in the same community positively impacts mental well-being in older adults through positive psychosocial mechanisms such as fostering a sense of belonging and attachment [12,13,81]. A moderating effect of social support on the influence of the number of asset-based initiatives on mental well-being was not observed, suggesting that other mechanisms not captured by our measure of perceived social support may be at play. For example, the mental well-being benefits can be derived not only from receiving but also from providing social support to others by enhancing feelings of independence and usefulness [82].

Interestingly, we also observed a dose-response trend in adjusted multilevel models, where higher uptake levels, measured as the regional availability of asset-based initiatives, were associated with increased social support and mental well-being. In Health Sectors with several available initiatives mobilized through intersectoral collaboration networks, individuals might leave unsatisfying activities for more meaningful opportunities, as involvement in newly formed social connections is voluntary, unlike family ties [20]. Previous studies have shown that having multiple asset-based initiatives to choose from can tailor to the preferences of older adults [45,83], which might lead to a better fit between initiatives and individual needs, leading to better social support and mental well-being in Health Sectors with more initiatives. Additionally, having access to diverse community assets can help individuals find a group of peers that better aligns with their culture and values [13,84]. To increase the likelihood of developing successful initiatives at the local level, involving potential participants and communities at all stages of conceptualization and implementation of intersectoral collaboration, especially in selecting community assets for the initiatives, is critical [33,76].

Finally, our findings identified several individual-level factors negatively associated with social support and mental well-being. Among these factors, being non-Spanish had the largest negative effect on social support, suggesting more restricted social networks among immigrant populations, as shown in previous literature [85]. Older women and those experiencing economic strain had significantly lower estimated mental well-being scores, confirming previous studies suggesting that socioeconomic factors and gender disparities play crucial roles in influencing mental well-being among older adults [86,87].

More importantly, our findings revealed a strong negative association between severe limitations in daily activities and mental well-being after controlling for sociodemographic and contextual covariates, with estimated SWEMWBS scores almost 5 points lower on average compared to those with no limitations. While these findings are consistent with previous literature [87,88], the magnitude of this secondary finding in a representative sample of older adults using the SWEMWBS scale has not been previously reported and warrants attention.

While isolated frail older adults with limitations that impede their participation in social activities could benefit from these initiatives, they frequently remain unreached [36]. Local stakeholders should strive to improve outreach to more frail segments of the population by adopting multifaceted approaches, like mobilizing volunteer networks for outreach and improving transportation services [89]. The multiple social determinants of health that negatively impact social support and mental well-being require adopting an equity lens in developing national public health programs [37,90]. Ensuring equitable access for older population with low income, immigrants, and women through targeted strategies is crucial [36,81]. Additionally, regions with limited capacity to map community health assets require robust governmental support and targeted investments to bridge gaps in social infrastructure, ensuring all older adults can benefit from these interventions.

### Limitations

This study has limitations. The cross-sectional design limits our ability to establish causality or the temporal sequence of events, such as whether higher regional uptake of the public health strategy and subsequent exposure to asset-based initiatives led to improved social support and mental well-being. Therefore, higher levels of social support and mental well-being may have resulted from alternative mechanisms, such as greater social cohesion or better neighbourhood socioeconomic and infrastructure conditions, which could facilitate the successful implementation of intersectoral collaboration networks for asset-based initiatives [91]. However, the observed dose-response pattern and specificity of associations suggest causal characteristics, warranting further investigation in future research.

Our analysis focused on data from 2017 onward, using independent cross-sectional survey waves, which precluded comparisons with pre-2015 data and the use of longitudinal designs. However, by categorizing participants into Health Sector uptake groups based on year-specific data, we accounted for temporal differences in initiative uptake and their relationships with social support and mental well-being.

The estimated effect sizes for social support and mental well-being scores were small relative to the measurement scale ranges, potentially limiting clinical significance. However, from a population health perspective, these differences may still be meaningful [92]. The multilevel models showed limited explanatory power for social support, suggesting potential unmeasured factors and low variability in social support scores, possibly reflecting selection bias as older adults with higher age or chronic conditions reported social support less frequently. Marital status was not included as a variable in the analysis due to its removal from the ESCA survey items in earlier waves, which may have contributed to unmeasured confounding. Additionally, Health Sector covariates for population density and centrality were unavailable, as the boundaries of these jurisdictions encompass a mix of rural and urban areas and do not align with standard census units. However, the inclusion of random effects for Health Sector in the multilevel models accounted for sector-level variability and unobserved differences, while the standardization of independent variables and the inclusion of contextual covariates to control for Health Sector population size, helped adjust for population differences across sectors.

We measured public health strategy uptake by assessing the area-level number and territorial distribution of initiatives, assuming uniform access to interventions within Health Sectors. Although multilevel models allow for the simultaneous analysis of contextual variables and individual outcomes, the generalizability of these contextual effects to the individual level remains debated [93,94]. To enhance the future evaluation of similar strategies, systematic monitoring and registration of individual use of intersectoral asset-based initiatives at the population level are recommended, supporting more robust longitudinal and experimental studies.

Finally, the number and territorial distribution of intersectoral asset-based initiatives were extracted from the Assets and Health platform, a government repository. Although robust data analytics methods were used, the actual number of initiatives may be underreported if local stakeholders do not consistently use the national repository. Limited identification and registration of health assets may be influenced by local priorities, management support, and socioeconomic context, as well as the coexistence of other local systems like shared Excel sheets or municipal mapping tools such as the Barcelona health search tool.

## Conclusion

This study examined the relationship between regional uptake of a national public health strategy and social support and mental well-being among older adults, using non-traditional data analytic techniques combined with survey data, highlighting the role of alternative data sources in public health research. Living in regions with a higher number of community health assets within intersectoral collaboration networks was associated with greater social support and mental well-being among older adults. These findings underscore the potential of local intersectoral collaboration to mobilize community health assets by identifying at-risk older adults, connecting them with social engagement opportunities, and implementing follow-up strategies. National public health strategies can support these efforts by providing training and logistical support to map and activate existing community assets. However, systematic monitoring and active efforts are crucial to ensure equitable availability of these initiatives across different regions.

## Supporting information

S1 File Characteristics of the asset-based initiatives and availability across jurisdictions. (DOCX)

S2 File Multilevel linear regressions. Full model outputs. (DOCX)

S3 File Sensitivity analyses. (DOCX)

## Abbreviations:

ESCA: Catalan Health Survey [Enquesta de Salut de Catalunya]
OSSS-3: Oslo Social Support Scale
SWEMWBS: Short Warwick-Edinburgh Mental Well-being Scale
