# Supplementary material for: Mobilizing community health assets through intersectoral collaboration for social connection: Associations with social support and well-being in a nationwide population-based study in Catalonia
Source: PLoS One. 2025 Mar 26;20(3):e0320317. doi: 10.1371/journal.pone.0320317 (PMC11940711; doi:10.1371/journal.pone.0320317)
Supplement: S1 File — (DOCX) [file pone.0320317.s001.docx]

**S1 File.** **Characteristics of the asset-based initiatives and availability across jurisdictions.**

Table S1. Characteristics of the asset-based intersectoral initiatives with potential to enhance older adults’ social connections.

| **New categories for activities characteristics N=2312** | **n (%)** |
| --- | --- |
|  |  |
| **Type of activity** |  |
| Leisure and skill development | 987 (42.7) |
| Physical activity | 674 (29.1) |
| Psychological therapies | 237 (10.25) |
| Health and social care | 210 (9.1) |
| Social facilitation | 154 (6.7) |
| Awareness campaigns | 39 (1.7) |
| Befriending | 5 (0.2) |
| Animal based | 6 (0.2) |
| **Format** |  |
| Group | 2275 (98.4) |
| Individual | 37 (1.6) |

Table S2. Definitions for each type of activity and category

| **Asset-based initiative type** | **Definitions and examples** |
| --- | --- |
| Leisure and skill development | Leisure activities are recreational activities such as gardening, travelling, playing, or dancing. Skill development activities focus on acquiring or improving skills, such as computer programming, cooking classes, or learning a new language. |
| Physical activity | Group activities involving exercise or leisure include group walking and adapted physical exercise for older adults. |
| Social facilitation | Social facilitation activities promote social interaction through group-based activities like charity-funded friendship clubs, reading groups, shared interest groups, daycare centres, and friendship enrichment programs. |
| Psychological therapies | These activities employ therapeutic techniques administered by trained professionals, often using facilitated group activities. Examples include humor therapy, mindfulness and stress reduction, reminiscence group therapy, and cognitive enhancement and social support interventions. |
| Awareness campaigns | Activities designed to enhance knowledge of health and social issues concerning older adults address problems like social isolation and loneliness among both healthcare professionals and the public. |
| Health and social care | Group activities where healthcare and social professionals support older adults through direct intervention or education include health education (e.g., seminars for older adults with diabetes), health promotion and prevention (e.g., fall prevention groups), and social care (e.g., literacy programs for older migrants). |
| Befriending | Befriending activities involve one-to-one interactions with volunteers over time, such as companion programs. |
| Animal-based | Activities involving animals, focused mainly on animal-assisted therapy. |
| **Format** | **Definitions and examples** |
| Group | Group activities involve two or more individuals. They can be led by a health professional or instructor, such as fitness classes or group therapy sessions, or be more informal, such as social gatherings, walking clubs, or reading clubs. |
| Individual | These activities are directed to one individual. Examples include individual therapies and one-to-one companionship activities, such as befriending.. |

Table S3. Cumulative number of initiatives per Health Sector (absolute counts, unstandardized), 2017-2021.

| Health Sector code | 2017 | 2018 | 2019 | 2020 | 2021 |
| --- | --- | --- | --- | --- | --- |
| 1 | 0 | 37 | 83 | 108 | 129 |
| 2 | 0 | 9 | 28 | 62 | 127 |
| 3 | 0 | 0 | 3 | 4 | 5 |
| 4 | 0 | 0 | 23 | 53 | 88 |
| 5 | 0 | 0 | 5 | 47 | 55 |
| 6 | 0 | 0 | 0 | 0 | 0 |
| 7 | 0 | 12 | 19 | 25 | 44 |
| 8 | 0 | 10 | 38 | 55 | 83 |
| 9 | 0 | 0 | 7 | 25 | 45 |
| 10 | 0 | 32 | 44 | 50 | 64 |
| 11 | 0 | 1 | 1 | 1 | 6 |
| 12 | 0 | 4 | 11 | 17 | 18 |
| 13 | 0 | 4 | 5 | 9 | 9 |
| 14 | 0 | 3 | 7 | 7 | 7 |
| 15 | 0 | 1 | 1 | 1 | 1 |
| 16 | 0 | 0 | 5 | 5 | 6 |
| 17 | 0 | 2 | 5 | 13 | 14 |
| 18 | 0 | 1 | 2 | 9 | 10 |
| 19 | 6 | 19 | 20 | 27 | 27 |
| 20 | 0 | 1 | 3 | 5 | 9 |
| 21 | 0 | 29 | 88 | 122 | 146 |
| 22 | 3 | 21 | 37 | 43 | 111 |
| 23 | 1 | 5 | 28 | 53 | 71 |
| 24 | 0 | 5 | 42 | 55 | 79 |
| 25 | 0 | 3 | 12 | 26 | 31 |
| 26 | 4 | 42 | 195 | 240 | 342 |
| 28 | 0 | 2 | 83 | 133 | 164 |
| 29 | 0 | 4 | 44 | 47 | 105 |
| 30 | 4 | 15 | 36 | 59 | 66 |
| 31 | 3 | 53 | 133 | 181 | 242 |
| 32 | 0 | 37 | 130 | 157 | 208 |

Table S4. Territorial reach, as proportion of the sub-jurisdictions within each Health Sector that registered one or more asset-based initiatives, 2017-2021.

| Health Sector code | 2017 | 2018 | 2019 | 2020 | 2021 |
| --- | --- | --- | --- | --- | --- |
| 1 | 0.00 | 0.30 | 0.60 | 0.65 | 0.65 |
| 2 | 0.00 | 0.25 | 0.75 | 0.75 | 0.88 |
| 3 | 0.00 | 0.00 | 0.18 | 0.18 | 0.18 |
| 4 | 0.00 | 0.00 | 0.43 | 0.43 | 0.71 |
| 5 | 0.00 | 0.00 | 0.56 | 0.67 | 0.67 |
| 6 | 0.00 | 0.00 | 0.00 | 0.00 | 0.00 |
| 7 | 0.00 | 0.31 | 0.38 | 0.38 | 0.54 |
| 8 | 0.00 | 0.33 | 0.53 | 0.53 | 0.67 |
| 9 | 0.00 | 0.00 | 0.29 | 0.29 | 0.29 |
| 10 | 0.00 | 0.43 | 0.50 | 0.57 | 0.71 |
| 11 | 0.00 | 0.40 | 0.40 | 0.40 | 0.60 |
| 12 | 0.00 | 0.30 | 0.60 | 0.70 | 0.80 |
| 13 | 0.00 | 0.38 | 0.50 | 0.50 | 0.50 |
| 14 | 0.00 | 0.33 | 0.33 | 0.33 | 0.33 |
| 15 | 0.00 | 0.20 | 0.20 | 0.20 | 0.20 |
| 16 | 0.00 | 0.00 | 0.20 | 0.20 | 0.20 |
| 17 | 0.00 | 0.14 | 0.29 | 0.29 | 0.29 |
| 18 | 0.00 | 0.12 | 0.25 | 0.25 | 0.38 |
| 19 | 0.67 | 0.83 | 1.00 | 1.00 | 1.00 |
| 20 | 0.00 | 0.10 | 0.20 | 0.20 | 0.30 |
| 21 | 0.00 | 0.26 | 0.49 | 0.63 | 0.63 |
| 22 | 0.15 | 0.54 | 0.54 | 0.62 | 0.85 |
| 23 | 0.17 | 0.33 | 0.50 | 0.67 | 0.67 |
| 24 | 0.00 | 0.43 | 0.57 | 0.64 | 0.64 |
| 25 | 0.00 | 0.20 | 0.27 | 0.47 | 0.53 |
| 26 | 0.17 | 0.50 | 0.75 | 0.75 | 0.88 |
| 28 | 0.00 | 0.18 | 0.27 | 0.27 | 0.36 |
| 29 | 0.00 | 0.17 | 0.33 | 0.44 | 0.61 |
| 30 | 0.18 | 0.36 | 0.64 | 0.82 | 0.82 |
| 31 | 0.05 | 0.16 | 0.16 | 0.22 | 0.43 |
| 32 | 0.00 | 0.21 | 0.53 | 0.58 | 0.74 |
