# Supplementary material for: Mobilizing community health assets through intersectoral collaboration for social connection: Associations with social support and well-being in a nationwide population-based study in Catalonia
Source: PLoS One. 2025 Mar 26;20(3):e0320317. doi: 10.1371/journal.pone.0320317 (PMC11940711; doi:10.1371/journal.pone.0320317)
Supplement: S2 File — (DOCX) [file pone.0320317.s002.docx]

**S2 File. Multilevel linear regressions. Full model outputs**

Table S5. Multilevel regression models for social support (OSSS-3), including main independent variable “Number of initiatives per 10,000 population”, adjusted for individual and contextual covariates.

|  | **Model 1** | **p-value** | **Model 2** | **p-value** | **Model 3** | **p-value** | **Model 4** | **p-value** |
| --- | --- | --- | --- | --- | --- | --- | --- | --- |
| **Fixed effects** | Coefficient (CI) |  | Coefficient (CI) |  | Coefficient (CI) |  | Coefficient (CI) |  |
| (Intercept) | 11.15 (10.99 – 11.30) | <0.001 | 11.38 (11.16 – 11.61) | <0.001 | 11.37 (11.14 – 11.59) | <0.001 | 11.30 (11.14 – 11.45) | <0.001 |
| **Main predictor** |  |  |  |  |  |  |  |  |
| Number of initiatives per 10,000 population |  |  |  |  |  |  |  |  |
| No uptake | ref |  | ref |  | ref |  | ref |  |
| 1-15 initiatives | 0.25 (0.05 – 0.44) | 0.013 | 0.25 (0.05 – 0.44) | 0.013 | 0.26 (0.07 – 0.46) | 0.009 | 0.25 (0.05 – 0.44) | 0.012 |
| > 15 initiatives | 0.32 (0.06 – 0.59) | 0.018 | 0.34 (0.07 – 0.60) | 0.014 | 0.37 (0.10 – 0.64) | 0.008 | 0.34 (0.07 – 0.60) | 0.013 |
| **Individual level covariates** |  |  |  |  |  |  |  |  |
| Age |  |  | 0.00 (-0.00 – 0.01) | 0.295 | 0.00 (-0.00 – 0.01) | 0.231 |  |  |
| Gender |  |  |  |  |  |  |  |  |
| Man |  |  | ref |  | ref |  |  |  |
| Woman |  |  | 0.02 (-0.08 – 0.12) | 0.723 | 0.02 (-0.09 – 0.12) | 0.738 |  |  |
| Chronic conditions |  |  | -0.05 (-0.07 – -0.03) | <0.001 | -0.05 (-0.07 – -0.03) | <0.001 | -0.05 (-0.06 – -0.03) | <0.001 |
| Limitation daily activities |  |  |  |  |  |  |  |  |
| No limitation |  |  | ref |  | ref |  | ref |  |
| Limitation, not severe |  |  | -0.07 (-0.19 – 0.05) | 0.278 | -0.07 (-0.19 – 0.06) | 0.287 | -0.06 (-0.18 – 0.06) | 0.358 |
| Severe limitation |  |  | -0.38 (-0.56 – -0.21) | <0.001 | -0.38 (-0.56 – -0.21) | <0.001 | -0.36 (-0.53 – -0.19) | <0.001 |
| Household members |  |  | 0.11 (0.06 – 0.15) | <0.001 | 0.11 (0.06 – 0.16) | <0.001 | 0.10 (0.06 – 0.15) | <0.001 |
| Education |  |  |  |  |  |  |  |  |
| Primary or less |  |  | ref |  | ref |  |  |  |
| Secondary or higher |  |  | -0.04 (-0.15 – 0.06) | 0.424 | -0.05 (-0.15 – 0.06) | 0.358 |  |  |
| Employment |  |  |  |  |  |  |  |  |
| Active |  |  | ref |  | ref |  |  |  |
| Retired or unemployed |  |  | -0.06 (-0.22 – 0.10) | 0.457 | -0.06 (-0.21 – 0.10) | 0.477 |  |  |
| Domestic work |  |  | -0.10 (-0.30 – 0.10) | 0.320 | -0.10 (-0.30 – 0.10) | 0.326 |  |  |
| Economic strain |  |  |  |  |  |  |  |  |
| No |  |  | ref |  | ref |  | ref |  |
| Yes |  |  | -0.18 (-0.31 – -0.05) | 0.007 | -0.18 (-0.31 – -0.05) | 0.008 | -0.18 (-0.31 – -0.05) | 0.007 |
| Nationality |  |  |  |  |  |  |  |  |
| National |  |  | ref |  | ref |  | ref |  |
| Non-national |  |  | -0.85(-1.12 – -0.58) | <0.001 | -0.85(-1.12 – -0.58) | <0.001 | -0.87 (-1.13 – -0.60) | <0.001 |
| **Survey year** |  |  |  |  |  |  |  |  |
| 2017 | ref |  | ref |  | ref |  | ref |  |
| 2018 | -0.38 (-0.56 – -0.20) | <0.001 | -0.46 (-0.65 – -0.28) | <0.001 | -0.47 (-0.65 – -0.29) | <0.001 | -0.46 (-0.64 – -0.28) | <0.001 |
| 2019 | -0.72 (-0.93 – -0.51) | <0.001 | -0.78 (-0.98 – -0.57) | <0.001 | -0.79 (-1.00 – -0.59) | <0.001 | -0.77 (-0.98 – -0.57) | <0.001 |
| 2020 | -0.69 (-0.92 – -0.45) | <0.001 | -0.74 (-0.97 – -0.51) | <0.001 | -0.76 (-0.99 – -0.53) | <0.001 | -0.74 (-0.97 – -0.51) | <0.001 |
| 2021 | -0.41 (-0.64 – -0.18) | <0.001 | -0.47 (-0.70 – -0.25) | <0.001 | -0.49 (-0.72 – -0.27) | <0.001 | -0.47 (-0.70 – -0.25) | <0.001 |
| **Contextual covariables** |  |  |  |  |  |  |  |  |
| HS % population over 75 years living alone |  |  |  |  | 0.00 (-0.04 – 0.05) | 0.908 |  |  |
| HS socioeconomic index |  |  |  |  | 0.01 (-0.00 – 0.02) | 0.123 |  |  |
| HS total population |  |  |  |  | -0.01 (-0.14– 0.13) | 0.912 |  |  |
| **Random Effects** |  |  |  |  |  |  |  |  |
| σ^2^ | 3.34 |  | 3.27 |  | 3.27 |  | 3.26 |  |
| τ_00_ _Health_Sector_ | 0.09 |  | 0.08 |  | 0.08  τ00 Health_Sector 0.08  ICC 0.02  N Health_Sector 31 |  | 0.08  τ00 Health_Sector 0.08  ICC 0.02  N Health_Sector 31 |  |
| ICC | 0.03 |  | 0.03 |  | 0.03 |  | 0.03 |  |
| N _Health_Sector_ | 31 |  | 31 |  | 31 |  | 31 |  |
| Observations | 6011 |  | 6011 |  | 6011 |  | 6011 |  |
| AIC | 24,397 |  | 24,323 |  | 24,344 |  | 24,291 |  |
| Marginal R^2^ / Conditional R^2^ | 0.012 / 0.037 |  | 0.036 / 0.060 |  | 0.037 / 0.062 |  | 0.035 / 0.060 |  |

Model 1 = regional program uptake variables, controlled for survey year, and Health Sectors; Model 2 = main exposure variable, controlled for individual covariates, survey year, and Health Sectors; Model 3 = same variables plus context-level covariates; Model 4 = parsimonious model. Coefficients denote the estimated differences in the average perceived social support score measured with the OSSS-3, which ranges from 3 to 14 points. σ2=within-group variance; τ00= between-group variance; ICC= Intraclass Correlation Coefficient; AIC= Akaike Information Criterion.

Table S6. Multilevel regression models for social support (OSSS-3), including main independent variable “Territorial distribution”, adjusted for individual and contextual covariates.

|  | **Model 1** | **p-value** | **Model 2** | **p-value** | **Model 3** | **p-value** | **Model 4** | **p-value** |
| --- | --- | --- | --- | --- | --- | --- | --- | --- |
| **Fixed effects** | Coefficient (CI) |  | Coefficient (CI) |  | Coefficient (CI) |  | Coefficient (CI) |  |
| (Intercept) | 11.15 (10.99 – 11.30) | <0.001 | 11.38 (11.15 – 11.60) | <0.001 | 11.36 (11.13 – 11.59) | <0.001 | 11.29 (11.14 – 11.45) | <0.001 |
| **Main predictor** |  |  |  |  |  |  |  |  |
| Territorial distribution |  |  |  |  |  |  |  |  |
| No uptake |  |  | ref |  | ref |  | ref |  |
| Lower than 50% | 0.25 (0.05 – 0.44) | 0.015 | 0.25 (0.05 – 0.44) | 0.013 | 0.26 (0.07 – 0.46) | 0.009 | 0.25 (0.05 – 0.44) | 0.012 |
| Higher than 50% | 0.28 (0.02 – 0.54) | 0.037 | 0.31 (0.05 – 0.57) | 0.019 | 0.35 (0.08 – 0.62) | 0.010 | 0.32 (0.06 – 0.58) | 0.018 |
| **Individual level covariates** |  |  |  |  |  |  |  |  |
| Age |  |  | 0.00 (-0.00 – 0.01) | 0.296 | 0.00 (-0.00 – 0.01) | 0.320 |  |  |
| Gender |  |  |  |  |  |  |  |  |
| Man |  |  | ref |  | ref |  |  |  |
| Woman |  |  | 0.02 (-0.09 – 0.12) | 0.729 | 0.02 (-0.09 – 0.12) | 0.745 |  |  |
| Chronic conditions |  |  | -0.05 (-0.07 – -0.03) | <0.001 | -0.05 (-0.07 – -0.03) | <0.001 | -0.05 (-0.06 – -0.03) | <0.001 |
| Limitation daily activities |  |  |  |  |  |  |  |  |
| No limitation |  |  | ref |  | ref |  | ref |  |
| Limitation, not severe |  |  | -0.07 (-0.19 – 0.06) | 0.287 | -0.06 (-0.19 – 0.06) | 0.297 | -0.06 (-0.18 – 0.06) | 0.369 |
| Severe limitation |  |  | -0.38 (-0.56 – -0.21) | <0.001 | -0.38 (-0.56 – -0.21) | <0.001 | -0.36 (-0.53 – -0.19) | <0.001 |
| Household members |  |  | 0.11 (0.06 – 0.16) | <0.001 | 0.11 (0.06 – 0.16) | <0.001 | 0.10 (0.06 – 0.15) | <0.001 |
| Education |  |  |  |  |  |  |  |  |
| Primary or less |  |  | ref |  | ref |  |  |  |
| Secondary or higher |  |  | -0.04 (-0.15 – 0.06) | 0.422 | -0.05 (-0.15 – 0.06) | 0.360 |  |  |
| Employment |  |  |  |  |  |  |  |  |
| Active |  |  | ref |  | ref |  |  |  |
| Retired or unemployed |  |  | -0.06 (-0.22 – 0.10) | 0.460 | -0.06 (-0.21 – 0.10) | 0.480 |  |  |
| Domestic work |  |  | -0.10 (-0.30 – 0.10) | 0.330 | -0.10 (-0.29 – 0.10) | 0.337 |  |  |
| Economic strain |  |  |  |  |  |  |  |  |
| No |  |  | ref |  | ref |  | ref |  |
| Yes |  |  | -0.18 (-0.31 – -0.05) | 0.006 | -0.18 (-0.31 – -0.05) | 0.007 | -0.18 (-0.31 – -0.05) | 0.007 |
| Nationality |  |  |  |  |  |  |  |  |
| National |  |  | ref |  | ref |  | ref |  |
| Non-national |  |  | -0.85(-1.12 – -0.58) | <0.001 | -0.84(-1.12 – -0.57) | <0.001 | -0.86 (-1.13 – -0.59) | <0.001 |
| **Survey year** |  |  |  |  |  |  |  |  |
| 2017 | ref |  | ref |  | ref |  | ref |  |
| 2018 | -0.38 (-0.56 – -0.20) | <0.001 | -0.47 (-0.65 – -0.28) | <0.001 | -0.47 (-0.66 – -0.29) | <0.001 | -0.46 (-0.64 – -0.28) | <0.001 |
| 2019 | -0.72 (-0.93 – -0.50) | <0.001 | -0.78 (-0.99 – -0.57) | <0.001 | -0.80 (-1.02 – -0.59) | <0.001 | -0.78 (-0.99 – -0.56) | <0.001 |
| 2020 | -0.68 (-0.92 – -0.44) | <0.001 | -0.74 (-0.98 – -0.50) | <0.001 | -0.77 (-1.01 – -0.53) | <0.001 | -0.74 (-0.98 – -0.50) | <0.001 |
| 2021 | -0.39 (-0.62 – -0.16) | <0.001 | -0.47 (-0.70 – -0.23) | <0.001 | -0.49 (-0.73 – -0.26) | <0.001 | -0.47 (-0.70 – -0.23) | <0.001 |
| **Contextual variables** |  |  |  |  |  |  |  |  |
| HS % population over 75 years living alone |  |  |  |  | 0.00 (-0.05 – 0.05) | 0.981 |  |  |
| HS socioeconomic index |  |  |  |  | 0.01 (-0.00 – 0.02) | 0.131 |  |  |
| HS total population |  |  |  |  | -0.01 (-0.15– 0.13) | 0.872 |  |  |
| **Random Effects** |  |  |  |  |  |  |  |  |
| σ^2^ | 3.34 |  | 3.26 |  | 3.26 |  | 3.26 |  |
| τ_00_ _Health_Sector_ | 0.09 |  | 0.09 |  | 0.09  τ00 Health_Sector 0.08  ICC 0.02  N Health_Sector 31 |  | 0.09  τ00 Health_Sector 0.08  ICC 0.02  N Health_Sector 31 |  |
| ICC | 0.03 |  | 0.03 |  | 0.03 |  | 0.03 |  |
| N _Health_Sector_ | 31 |  | 31 |  | 31 |  | 31 |  |
| Observations | 6011 |  | 6011 |  | 6011 |  | 6011 |  |
| AIC | 24,398 |  | 24,324 |  | 24,345 |  | 24,291 |  |
| Marginal R^2^ / Conditional R^2^ | 0.012 / 0.038 |  | 0.035 / 0.061 |  | 0.037 / 0.063 |  | 0.035 / 0.061 |  |

Model 1 = regional program uptake variables, controlled for survey year, and Health Sectors; Model 2 = main exposure variable, controlled for individual covariates, survey year, and Health Sectors; Model 3 = same variables plus context-level covariates; Model 4 = parsimonious model. Coefficients denote the estimated differences in the average perceived social support score measured with the OSSS-3, which ranges from 3 to 14 points. σ2=within-group variance; τ00= between-group variance; ICC= Intraclass Correlation Coefficient; AIC= Akaike Information Criterion.

Table S7. Multilevel regression models for mental well-being (SWEMWBS), including main independent variable “Number of initiatives per 10,000 population”, adjusted for individual and contextual covariates.

|  | **Model 1** | **p-value** | **Model 2** | **p-value** | **Model 3** | **p-value** | **Model 4** | **p-value** |
| --- | --- | --- | --- | --- | --- | --- | --- | --- |
| **Fixed effects** | Coefficient (CI) |  | Coefficient (CI) |  | Coefficient (CI) |  | Coefficient (CI) |  |
| (Intercept) | 28.34 (27.88 – 28.80) | <0.001 | 29.80 (29.17 – 30.43) | <0.001 | 29.79 (29.15 – 30.43) | <0.001 | 29.82 (29.34 – 30.30) | <0.001 |
| **Main predictors** |  |  |  |  |  |  |  |  |
| Number of initiatives per 10,000 population |  |  |  |  |  |  |  |  |
| No uptake | ref |  | ref |  | ref |  | ref |  |
| 1-15 initiatives | 0.40 (-0.22 – 1.03) | 0.208 | 0.58 (0.03 – 1.13) | 0.040 | 0.59 (0.03 – 1.14) | 0.040 | 0.59 (0.04 – 1.14) | 0.036 |
| > 15 initiatives | 0.84 (-0.01 – 1.70) | 0.054 | 1.10 (0.35 – 1.86) | 0.004 | 1.11 (0.34 – 1.87) | 0.005 | 1.11 (0.36 – 1.86) | 0.004 |
| **Individual level covariates** |  |  |  |  |  |  |  |  |
| Age |  |  | -0.03(-0.05 – -0.02) | <0.001 | -0.03(-0.05 – -0.02) | <0.001 | -0.03(-0.05 – -0.02) | <0.001 |
| Gender |  |  |  |  |  |  |  |  |
| Man |  |  | ref |  | ref |  | ref |  |
| Woman |  |  | -0.81 (-1.11 – -0.52) | <0.001 | -0.81 (-1.11 – -0.52) | <0.001 | -0.90 (-1.17 – -0.64) | <0.001 |
| Chronic conditions |  |  | -0.38 (-0.43 – -0.33) | <0.001 | -0.38 (-0.43 – -0.33) | <0.001 | -0.38 (-0.43 – -0.33) | <0.001 |
| Limitation daily activities |  |  |  |  |  |  |  |  |
| No limitation |  |  | ref |  | ref |  | ref |  |
| Limitation, not severe |  |  | -2.16 (-2.51 – -1.81) | <0.001 | -2.15 (-2.50 – -1.80) | <0.001 | -2.16 (-2.51 – -1.81) | <0.001 |
| Severe limitation |  |  | -4.94 (-5.43 – -4.44) | <0.001 | -4.93 (-5.43 – -4.44) | <0.001 | -4.92 (-5.42 – -4.43) | <0.001 |
| Household members |  |  | 0.04 (-0.10 – 0.18) | 0.553 | 0.04 (-0.10 – 0.18) | 0.558 |  |  |
| Education |  |  |  |  |  |  |  |  |
| Primary or less |  |  | ref |  | ref |  | ref |  |
| Secondary or higher |  |  | 0.76 (0.46 – 1.05) | <0.001 | 0.75 (0.45 – 1.05) | <0.001 | 0.77 (0.47 – 1.06) | <0.001 |
| Employment |  |  |  |  |  |  |  |  |
| Active |  |  | ref |  | ref |  |  |  |
| Retired or unemployed |  |  | 0.04 (-0.41 – 0.49) | 0.853 | 0.05 (-0.41 – 0.50) | 0.842 |  |  |
| Domestic work |  |  | -0.22 (-0.79 – 0.34) | 0.438 | -0.22 (-0.79 – 0.34) | 0.441 |  |  |
| Economic strain |  |  |  |  |  |  |  |  |
| No |  |  | ref |  | ref |  | ref |  |
| Yes |  |  | -1.13 (-1.50 – -0.75) | <0.001 | -1.12 (-1.50 – -0.75) | <0.001 | -1.13 (-1.50 – -0.76) | <0.001 |
| Nationality |  |  |  |  |  |  |  |  |
| National |  |  | ref |  | ref |  |  |  |
| Non-national |  |  | -0.07 (-0.84 – 0.71) | 0.863 | -0.06 (-0.84 – 0.72) | 0.880 |  |  |
| **Survey year** |  |  |  |  |  |  |  |  |
| 2017 | ref |  | ref |  | ref |  | ref |  |
| 2018 | -0.37 (-0.95 – 0.22) | 0.218 | -1.06 (-1.58 – -0.54) | <0.001 | -1.06 (-1.58 – -0.54) | <0.001 | -1.07 (-1.58 – -0.55) | <0.001 |
| 2019 | -0.64 (-1.31 – 0.02) | 0.057 | -1.11 (-1.70 – -0.52) | <0.001 | -1.11 (-1.71 – -0.52) | <0.001 | -1.11 (-1.70 – -0.52) | <0.001 |
| 2020 | -0.75 (-1.49 – 0.00) | 0.050 | -1.26 (-1.92 – -0.60) | <0.001 | -1.26 (-1.93 – -0.60) | <0.001 | -1.26 (-1.92 – -0.60) | <0.001 |
| 2021 | -0.66 (-1.39 – 0.06) | 0.073 | -1.24 (-1.88 – -0.59) | <0.001 | -1.24 (-1.89 – -0.58) | <0.001 | -1.23 (-1.87 – -0.59) | <0.001 |
| **Contextual variables** |  |  |  |  |  |  |  |  |
| HS % population over 75 years living alone |  |  |  |  | -0.02 (-0.14 – 0.10) | 0.795 |  |  |
| HS socioeconomic index |  |  |  |  | 0.01 (-0.02 – 0.04) | 0.547 |  |  |
| HS total population |  |  |  |  | 0.04 (-0.31 – 0.40) | 0.806 |  |  |
| **Random Effects** |  |  |  |  |  |  |  |  |
| σ^2^ | 34.89 |  | 26.87 |  | 26.87 |  | 26.86 |  |
| τ_00_ _Health_Sector_ | 0.68 |  | 0.52 |  | 0.58 |  | 0.53 |  |
| ICC | 0.02 |  | 0.02 |  | 0.02 |  | 0.02 |  |
| N _Health_Sector_ | 31 |  | 31 |  | 31 |  | 31 |  |
| Observations | 6011 |  | 6011 |  | 6011 |  | 6011 |  |
| AIC | 38476 |  | 36947 |  | 36965 |  | 36935 |  |
| Marginal R^2^ / Conditional R^2^ | 0.001 / 0.020 |  | 0.231 / 0.246 |  | 0.231 / 0.247 |  | 0.231 / 0.246 |  |

Model 1 = regional program uptake variables, controlled for survey year, and Health Sectors; Model 2 = main exposure variable, controlled for individual covariates, survey year, and Health Sectors; Model 3 = same variables plus context-level covariates; Model 4 = parsimonious model. Coefficients denote the estimated differences in the average mental well-being score measured with the SWEMWBS scale, which ranges from 7 to 35 points. σ2=within-group variance; τ00= between-group variance; ICC= Intraclass Correlation Coefficient; AIC= Akaike Information Criterion.

Table S8. Multilevel regression models for mental well-being (SWEMWBS), including main independent variable “Territorial distribution”, adjusted for individual and contextual covariates.

|  | **Model 1** | **p-value** | **Model 2** | **p-value** | **Model 3** | **p-value** | **Model 4** | **p-value** |
| --- | --- | --- | --- | --- | --- | --- | --- | --- |
| **Fixed effects** | Coefficient (CI) |  | Coefficient (CI) |  | Coefficient (CI) |  | Coefficient (CI) |  |
| (Intercept) | 28.33 (27.85 – 28.80) | <0.001 | 29.78 (29.14 – 30.42) | <0.001 | 29.78 (29.13 – 30.43) | <0.001 | 29.81 (29.31 – 30.30) | <0.001 |
| **Main predictors** |  |  |  |  |  |  |  |  |
| Territorial distribution |  |  |  |  |  |  |  |  |
| No uptake | ref |  | ref |  | ref |  | ref |  |
| Lower than 50% | 0.33 (-0.30 – 0.96) | 0.302 | 0.53 -0.03 – 1.08 | 0.064 | 0.52 (-0.04 – 1.09) | 0.070 | 0.54 (-0.02 – 1.10) | 0.058 |
| Higher than 50% | -0.16 (-1.00 – 0.68) | 0.717 | 0.33 -0.41 – 1.08 | 0.381 | 0.32 (-0.44 – 1.08) | 0.409 | 0.34 (-0.40 – 1.09) | 0.367 |
| **Individual level covariates** |  |  |  |  |  |  |  |  |
| Age |  |  | -0.03 (-0.05 – -0.02) | <0.001 | -0.03 (-0.05 – -0.02) | <0.001 | -0.03 (-0.05 – -0.02) | <0.001 |
| Gender |  |  |  |  |  |  |  |  |
| Man |  |  | ref |  | ref |  | ref |  |
| Woman |  |  | -0.81 (-1.11 – -0.52) | <0.001 | -0.81 (-1.11 – -0.52) | <0.001 | -0.90 (-1.17 – -0.64) | <0.001 |
| Chronic conditions |  |  | -0.38 (-0.43 – -0.33 )*** | <0.001 | -0.38 (-0.43 – -0.33) | <0.001 | -0.38 (-0.43 – -0.33) | <0.001 |
| Limitation daily activities |  |  |  |  |  |  |  |  |
| No limitation |  |  | ref |  | ref |  | ref |  |
| Limitation, not severe |  |  | -2.15 (-2.50 – -1.80) | <0.001 | -2.14 (-2.49 – -1.79) | <0.001 | -2.14 (-2.49 – -1.79) | <0.001 |
| Severe limitation |  |  | -4.92 (-5.42 – -4.43) | <0.001 | -4.92 (-5.42 – -4.42) | <0.001 | -4.91 (-5.41 – -4.42) | <0.001 |
| Household members |  |  | 0.04 (-0.10 – 0.18) | 0.585 | 0.04 (-0.10 – 0.18) | 0.592 |  |  |
| Education |  |  |  |  |  |  |  |  |
| Primary or less |  |  | ref |  | ref |  | ref |  |
| Secondary or higher |  |  | 0.74 (0.44 – 1.04) | <0.001 | 0.74 (0.44 – 1.04) | <0.001 | 0.75 (0.46 – 1.05) | <0.001 |
| Employment |  |  |  |  |  |  |  |  |
| Active |  |  | ref |  | ref |  |  |  |
| Retired or unemployed |  |  | 0.05 (-0.40 – 0.50) | 0.832 | 0.05 (-0.40 – 0.50) | 0.829 |  |  |
| Domestic work |  |  | -0.21 (-0.78 – 0.35) | 0.463 | -0.21 (-0.78 – 0.35) | 0.463 |  |  |
| Economic strain |  |  |  |  |  |  |  |  |
| No |  |  | ref |  | ref |  | ref |  |
| Yes |  |  | -1.13 (-1.50 – -0.76) | <0.001 | -1.13 (-1.51 – -0.76) | <0.001 | -1.13 (-1.51 – -0.76) | <0.001 |
| Nationality |  |  |  |  |  |  |  |  |
| National |  |  | ref |  | ref |  |  |  |
| Non-national |  |  | -0.04 (-0.82 – 0.73) | 0.911 | -0.04 (-0.81 – 0.74) | 0.925 |  |  |
| **Survey year** |  |  |  |  |  |  |  |  |
| 2017 | ref |  | ref |  | ref |  | ref |  |
| 2018 | -0.31 -0.89 – 0.28 | 0.307 | -1.02 (-1.54 – -0.50) | <0.001 | -1.02 (-1.54 – -0.50) | <0.001 | -1.03 (-1.55 – -0.51) | <0.001 |
| 2019 | -0.31 -1.00 – 0.37 | 0.372 | -0.88 (-1.49 – -0.27) | 0.005 | -0.87 (-1.49 – -0.26) | 0.006 | -0.88 (-1.49 – -0.27) | 0.005 |
| 2020 | -0.32 -1.09 – 0.46 | 0.421 | -0.96 (-1.65 – -0.28) | 0.006 | -0.95 (-1.65 – -0.26) | 0.007 | -0.96 (-1.65 – -0.28) | 0.006 |
| 2021 | -0.03 -0.78 – 0.72 | 0.933 | -0.76 (-1.43 – -0.10) | 0.024 | -0.76 (-1.43 – -0.08) | 0.028 | -0.76 (-1.43 – -0.10) | 0.025 |
| **Contextual variables** |  |  |  |  |  |  |  |  |
| HS % population over 75 years living alone |  |  |  |  | -0.03 (-0.16 – 0.10) | 0.683 |  |  |
| HS socioeconomic index |  |  |  |  | 0.00 (-0.03 – 0.04) | 0.862 |  |  |
| HS total population |  |  |  |  | 0.04 (-0.35 – 0.43) | 0.849 |  |  |
| **Random Effects** |  |  |  |  |  |  |  |  |
| σ^2^ | 34.86 |  | 26.86 |  | 26.86 |  | 26.85 |  |
| τ_00_ _Health_Sector_ | 0.78 |  | 0.65 |  | 0.73 |  | 0.66 |  |
| ICC | 0.02 |  | 0.02 |  | 0.03 |  | 0.02 |  |
| N _Health_Sector_ | 31 |  | 31 |  | 31 |  | 31 |  |
| Observations | 6011 |  | 6011 |  | 6011 |  | 6011 |  |
| AIC | 38475 |  | 36951 |  | 36968 |  | 36939 |  |
| Marginal R^2^ / Conditional R^2^ | 0.001 / 0.023 |  | 0.230 / 0.248 |  | 0.230 / 0.250 |  | 0.230 / 0.248 |  |

Model 1 = regional program uptake variables, controlled for survey year, and Health Sectors; Model 2 = main exposure variable, controlled for individual covariates, survey year, and Health Sectors; Model 3 = same variables plus context-level covariates; Model 4 = parsimonious model. Coefficients denote the estimated differences in the average mental well-being score measured with the SWEMWBS scale, which ranges from 7 to 35 points. σ2=within-group variance; τ00= between-group variance; ICC= Intraclass Correlation Coefficient; AIC= Akaike Information Criterion.
