# Supplementary material for: Mobilizing community health assets through intersectoral collaboration for social connection: Associations with social support and well-being in a nationwide population-based study in Catalonia
Source: PLoS One. 2025 Mar 26;20(3):e0320317. doi: 10.1371/journal.pone.0320317 (PMC11940711; doi:10.1371/journal.pone.0320317)

**S3 File. Sensitivity analyses.**

**Sensitivity analysis 1.** Parsimonious model with imputed missing values versus original dataset.

**
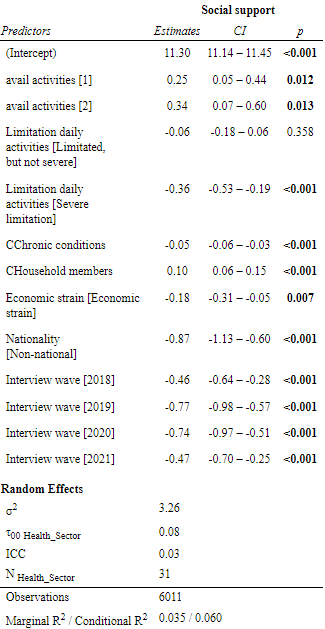
OSSS-3 ~ total initiatives. Imputed dataset OSSS-3 ~ total initiatives. NAs dataset
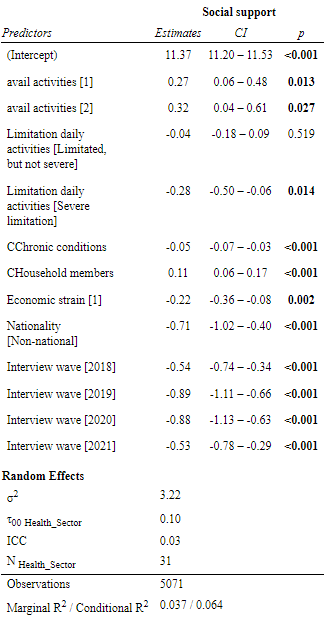
**

**
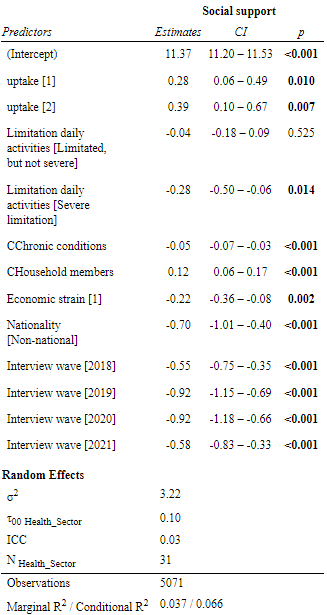

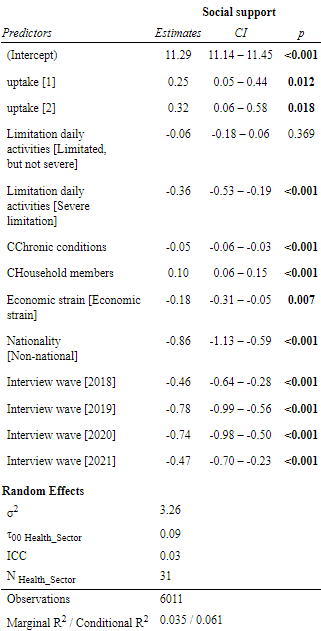
OSSS-3 ~ Territorial reach. Imputed dataset OSSS-3~ Territorial reach. NAs dataset**

**
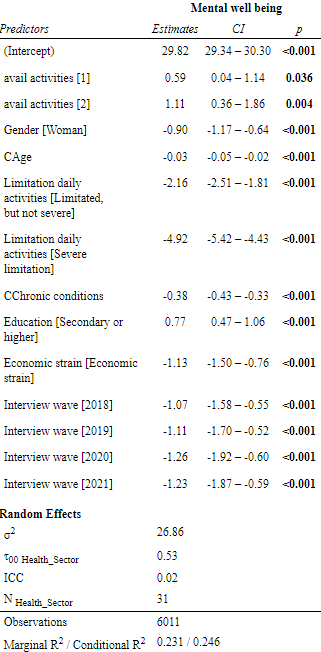
 SWEMWBS ~ total initiatives. Imputed dataset SWEMWBS ~ total initiatives. NAs dataset**

**
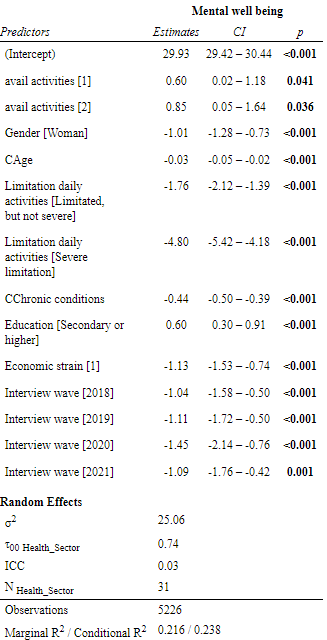
**

**SWEMWBS ~ Territorial reach. Imputed dataset SWEMWBS ~ Territorial reach. NAs dataset**

**
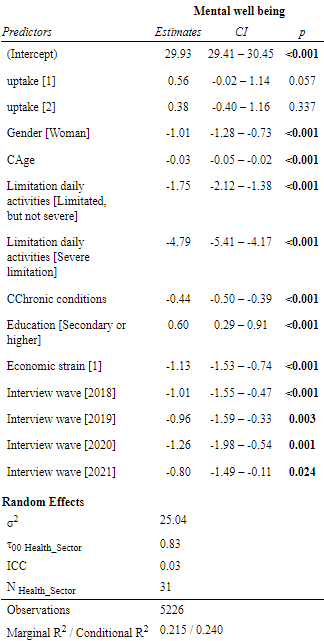

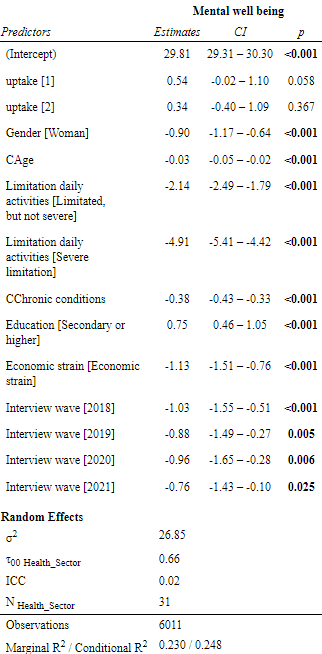
**

**Sensitivity analysis 2.** Parsimonious multilevel models versus standard linear regression with Health Sectors as fixed effects. (Health sector as fixed effects n=31 -1 dummy variables not shown)

**
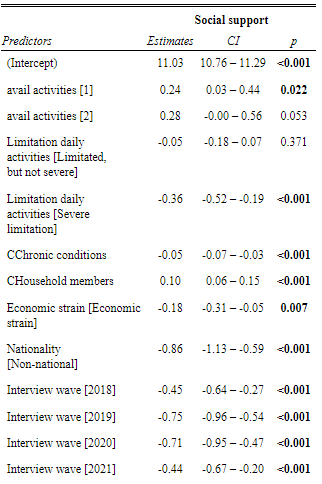
 OSSS-3 ~ total initiatives. Multilevel model** **OSSS-3 ~ total initiatives. Linear regression**

**
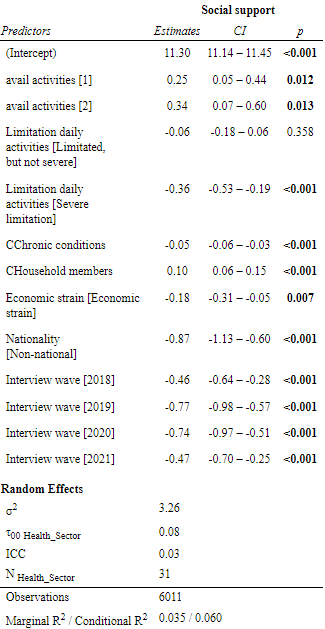
**

**
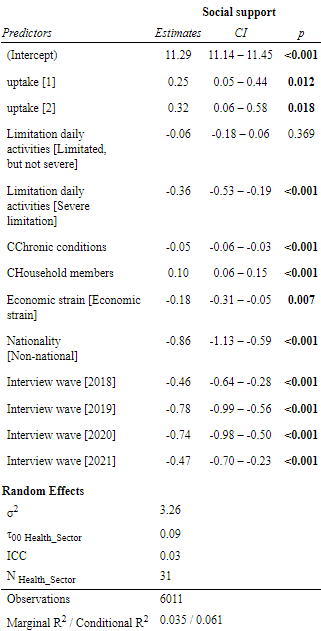
 OSSS-3 ~ territorial reach. Multilevel model OSSS-3 ~ territorial reach. Linear regression**

**
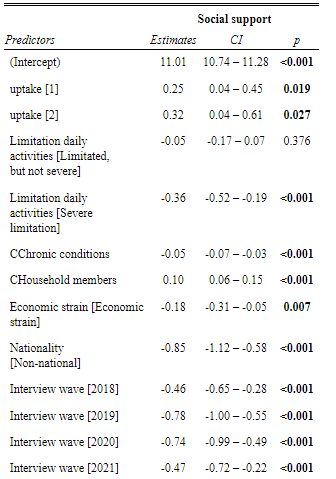
**

**SWEMWBS ~ total initiatives. Multilevel model SWEMWBS ~ total initiatives. Linear regression**

**
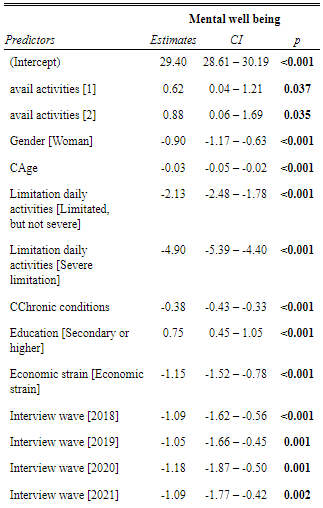

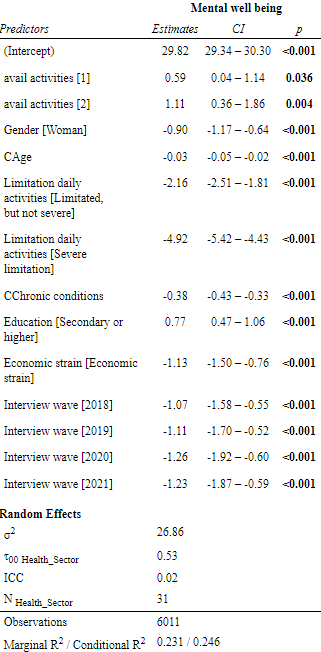
**

**Sensitivity analysis 3.** Parsimonious multilevel models adjusting for potential outliers with large influence using robust multilevel estimation.

*Testing and adjusting level-1 outliers: Robust multilevel models social support ~ total initiatives*

Upon re-evaluating the parsimonious models using robust multilevel regression, adjustments were made to a total of 1,176 outlier observations (19.5%) which commonly had lower social support scores. The high median weight assignment to the outliers of 0.81 (IQR = 0.25) indicates that most of these flagged observations might be moderate outliers.

| Robustness weights for the residuals: |
| --- |
| 4835 weights are ~= 1. The remaining 1176 ones are summarized as |
| Min. 1st Qu. Median Mean 3rd Qu. Max. |
| 0.313 0.671 0.814 0.783 0.925 0.999 |

**
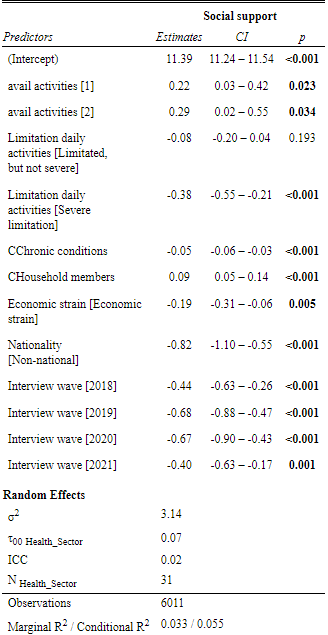

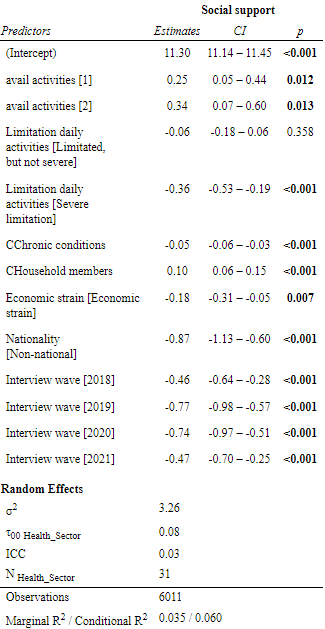
****Multilevel parsimonious model OSSS-3~ total initiatives Robust estimates**

*Testing and adjusting level-1 outliers: Robust multilevel models social support ~ territorial reach.*

| Robustness weights for the residuals: |
| --- |
| 4836 weights are ~= 1. The remaining 1175 ones are summarized as |
| Min. 1st Qu. Median Mean 3rd Qu. Max. |
| 0.314 0.668 0.813 0.783 0.925 0.999 |

**Multilevel parsimonious model OSSS-3 ~territorial reach Robust estimates**

*
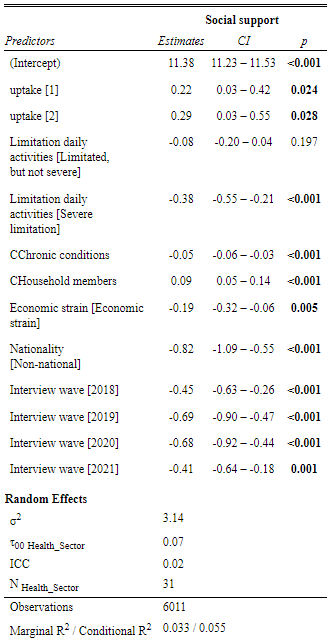

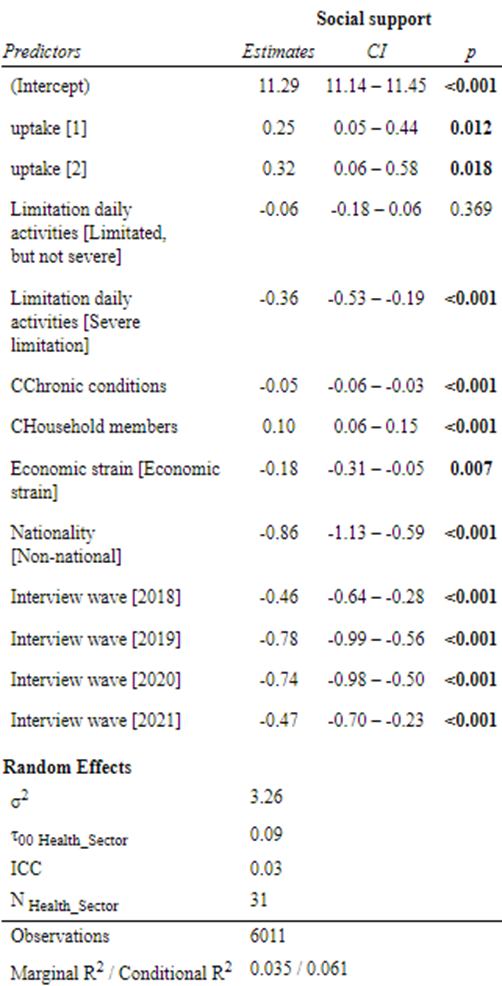
*

*Outliers at the random effects level (Health Sectors)*

No Health Sectors were observed to have a Cook’s distance >0.4, indicating no Health Sectors with a larger influence in the models.


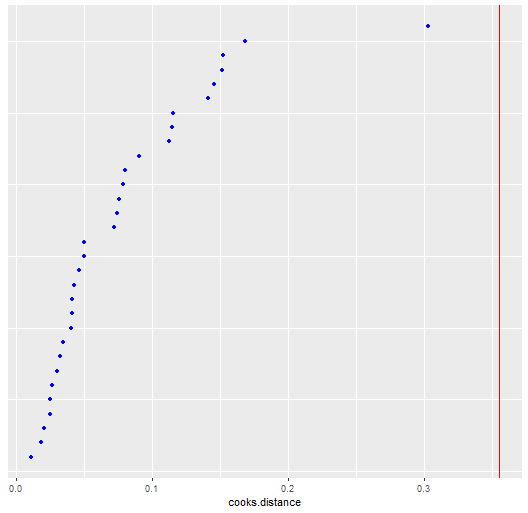


*Testing and adjusting level-1 outliers: Robust multilevel models mental well-being SWEMWBS ~ total initiatives*

A total of 1,117 outliers (18.6%) with moderate influence were adjusted for through robust estimations, yielding similar significant positive association.

| 4894 weights are ~= 1. The remaining 1117 ones are summarized as |
| --- |
| Min. 1st Qu. Median Mean 3rd Qu. Max. |
| 0.258 0.628 0.798 0.765 0.931 0.999 |
|  |


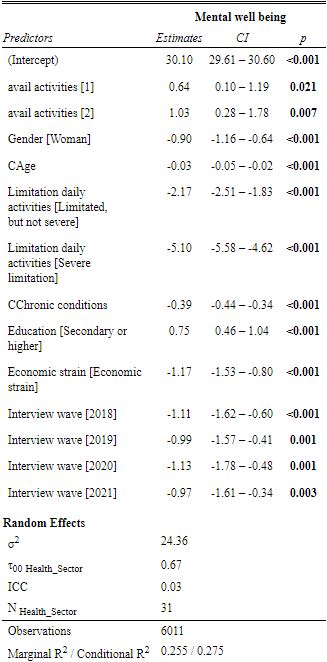
**
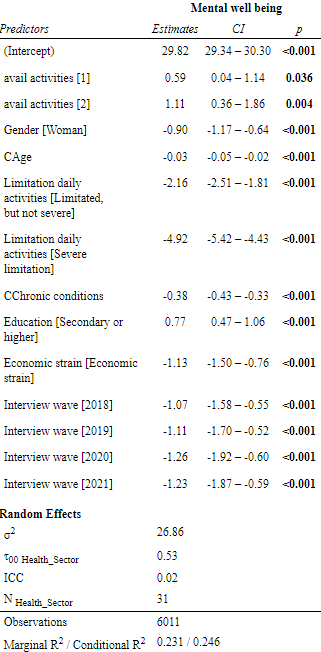
Multilevel parsimonious model SWEMWBS~ total initiatives Robust estimates**

*Outliers at the contextual level (Health Sectors)*


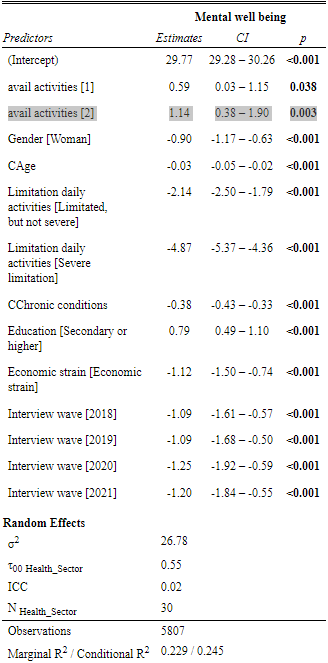

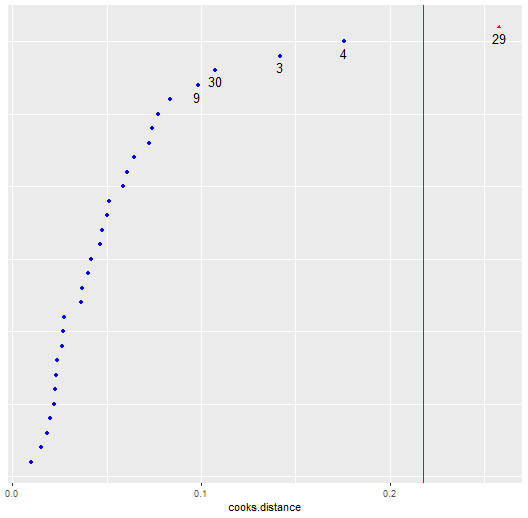
Health Sector 29 was identified as an outlier, a sensitivity analysis was conducted by removing this health sector. The resulting Model is attached below.

**Sensitivity analysis 4**. Robustness check. Outcomes as Ordinal (OSSS-3) and Dichotomous (SWEMWBS) variables

The sensitivity analysis with ordinal (OSSS-3) and dichotomous (SWEMWBS) outcomes for robustness check showed results with a stepwise increase in effect through Odds Ratios (OR), aligning with the original models’ results. Individuals residing in Health Sectors with 1-15 initiatives had 1.33 times greater odds of reporting higher social support (OSSS-3) (OR = 1.33, 95% CI = 1.08 to 1.63, p < 0.01), while those in sectors with more than 15 initiatives had 1.66 times greater odds (OR = 1.66, 95% CI = 1.25 to 2.19, p < 0.001), compared to those in Health Sectors with no initiatives. A similar stepwise trend were observed for mental well-being (SWEMWBS): 1-15 initiatives (OR = 1.31, 95% CI 1.01 to 1.70, P < .05); >15 initiatives (OR = 1.41, 95% CI 0.99 to 1.99, P = 0.054).

**Odds Ratio OSSS-3 (ordinal) ~ Total initiatives + covariates**


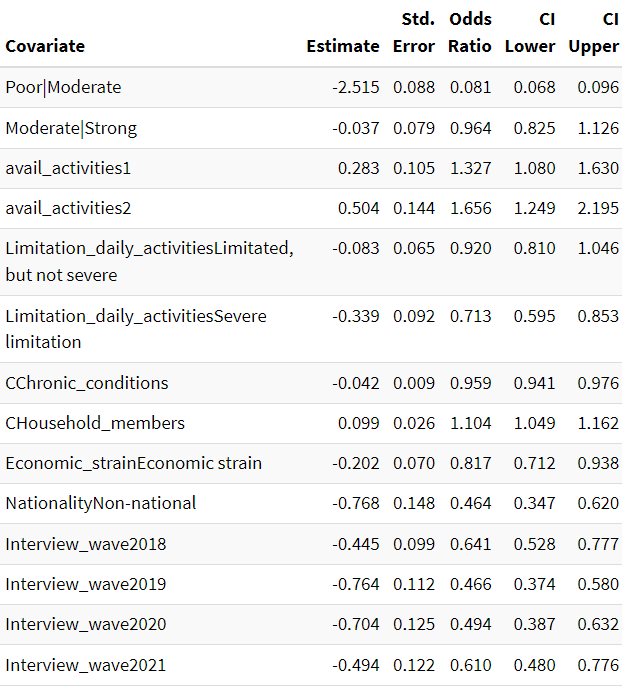


**Odds Ratio OSSS-3 (ordinal) ~ Territorial reach + covariates**


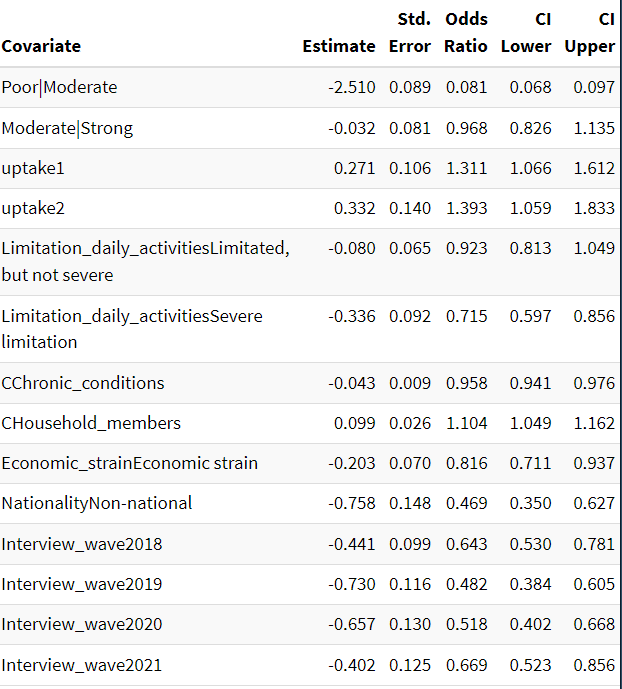


**Odds Ratio SWEMWBS (dichotomous) ~ Total initiatives + covariates**


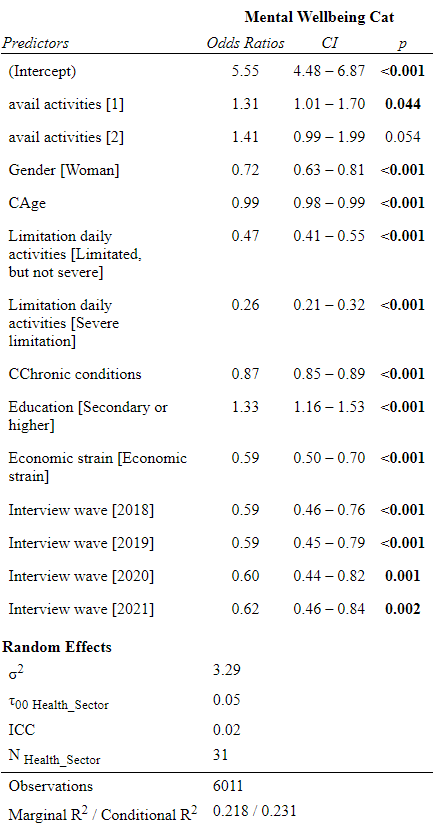


**Sensitivity analysis 5.** Specificity analysis. Chronic conditions as outcome

Specificity of associations was confirmed using the number of chronic conditions as an alternative outcome, showing no significant association between living in a Health Sector with a higher number of initiatives or greater territorial reach.

**Chronic conditions ~ total initiatives + covariates Chronic conditions ~ territorial reach +**

**covariates**


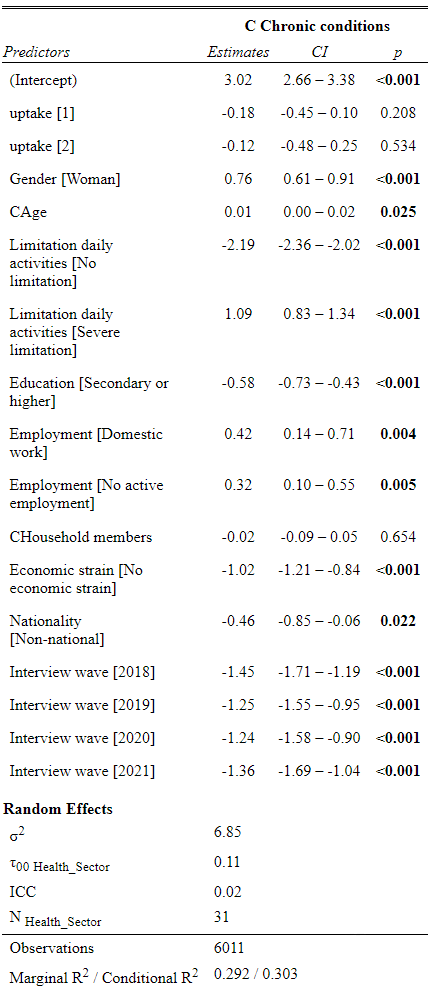

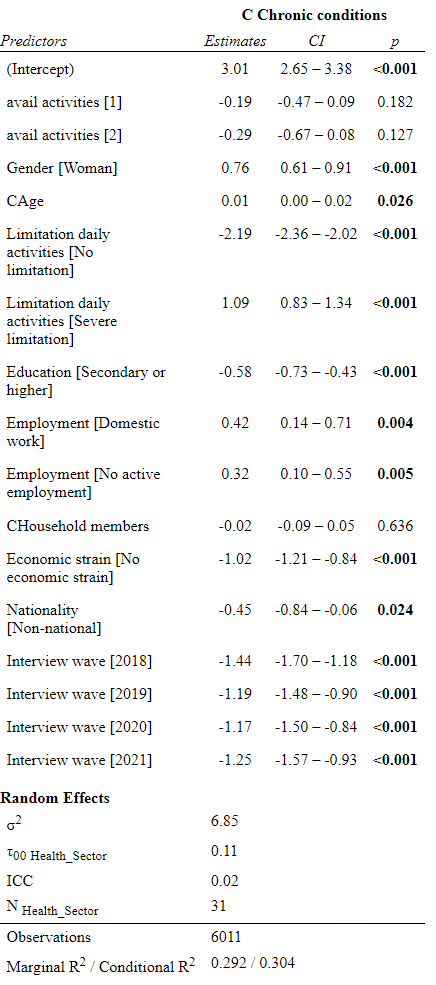


**Sensitivity analysis 6.** Impact of COVID-19 on models. Parsimonious models removing survey years 2020-2021.

Sensitivity analysis of the impact of the COVID-19 pandemic (removing survey years 2020-2021) revealed that the most exposed group reported significantly higher coefficients for living in Health Sectors with more than 15 initiatives compared to the original models. Compared to those in areas with no initiatives, higher estimates were found for social support scores (OSSS-3) (β = 0.47, 95% CI = 0.10–0.83, p < 0.05) and mental well-being (SWEMWBS) (β = 1.45, 95% CI = 0.41–2.49, p < 0.01).

**OSSS-3 ~ Total initiatives OSSS-3 ~ Territorial reach**


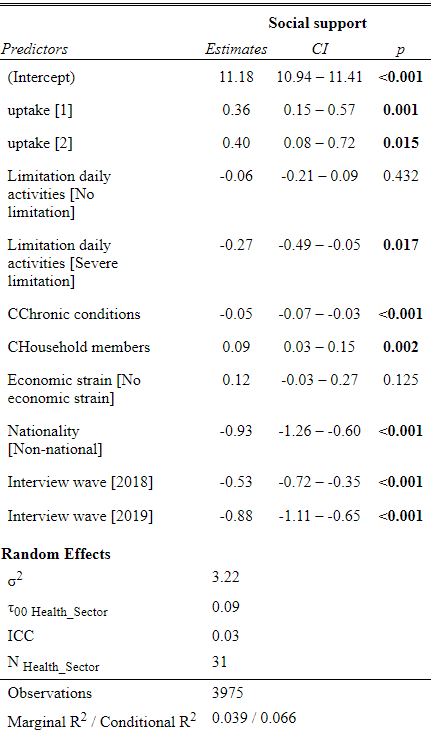

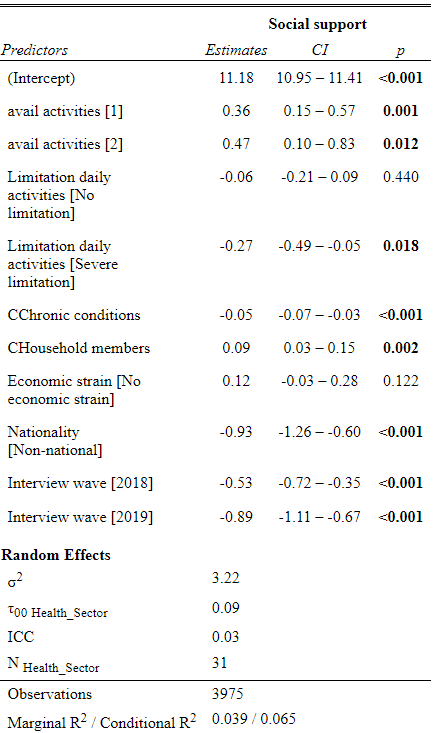


**SWEMWBS ~ total initiatives**


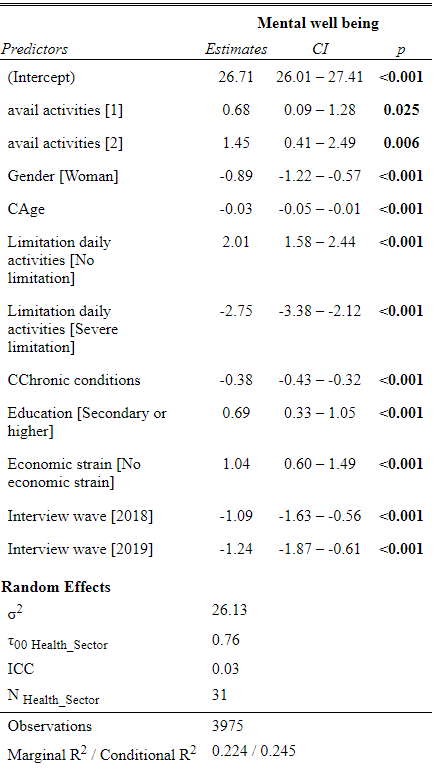


**Sensitivity analysis 7.** Parsimonious models with population subset >65 years

When analyzing populations over 65 years, the coefficients indicated similar effects to the original analysis (Supplementary File 3).

**OSSS-3 ~ Total initiatives OSSS-3 ~ Territorial reach**


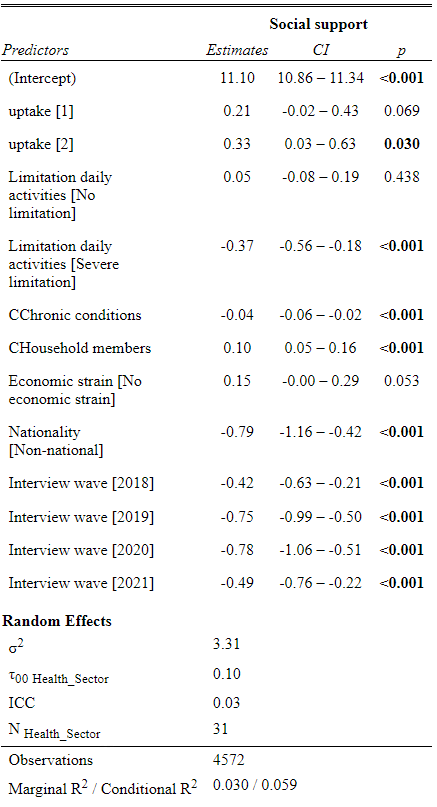

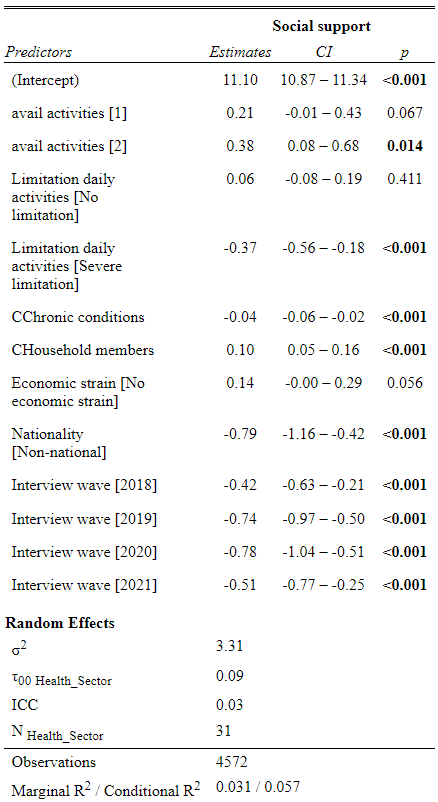


**SWEMWBS ~ total initiatives**


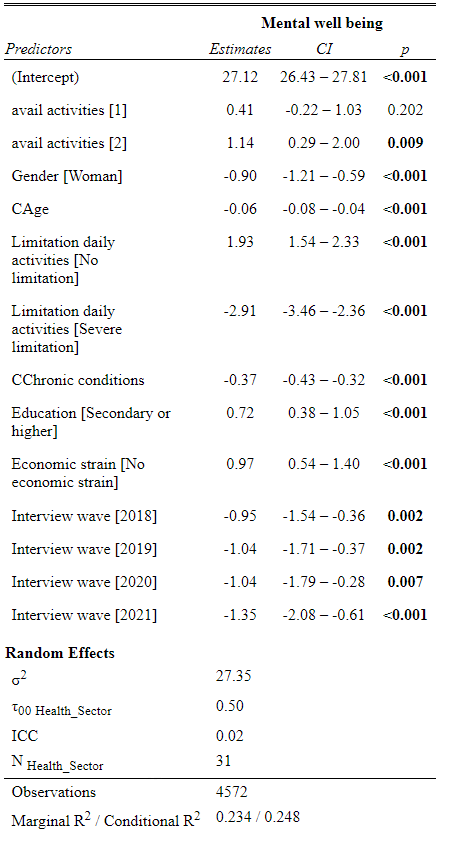

Supplement: S3 File — (DOCX) [file pone.0320317.s003.docx]
